# Synergistic effects of structured and powdered Calcium Chloride-Activated Carbon composites on Ammonia adsorption: the role of salt distribution and pH-controlled crosslinking

**DOI:** 10.1007/s42247-025-01106-8

**Published:** 2025-05-08

**Authors:** Sarah Farrukh, Xianfeng Fan, Syed Shujaat Karim, Zhibin Yu, Guanchu Lu

**Affiliations:** 1 School of Engineering, Institute for Materials and Processes, The University of Edinburgh, Edinburgh, Scotland, EH9 3 FB UK; 2 School of Chemical and Materials Engineering (SCME), National University of Sciences and Technology (NUST), Sector H-12, Islamabad, Pakistan; 3 Department of Mechanical and Aerospace Engineering, School of Engineering, The University of Liverpool, Liverpool, UK; 4 The Chinese University of Hong Kong, Shenzhen, China

**Keywords:** Ammonia adsorption, Hybrid fillers, Calcium chloride, Activated carbon, Kinetic analysis, Energy storage, Economic analysis

## Abstract

The porous powdered composites, containing metal halide salts are being used to enhance the storage of cold thermal energy storage (CTES) medium such as ammonia for efficient cooling systems. However, due to the low mass transfer attributable to agglomeration, reduction in pore accessibility, and sample loss, the powdered samples face challenges translating from lab to pilot scale. Conversely, the structuring of composites in beads and pallets can modify the surface chemistry and adsorption mechanism. To investigate the impact of structuring, a comparative analysis of pure, calcium chloride (CaCl_2_)-impregnated activated carbon (AC) powder and CaCl_2_-crosslinked AC beads was conducted in this work, to observe the ramification of surface chemistry due to salt distribution, porosity, surface area and pH-controlled crosslinking, on ammonia adsorption. The role of pH in bead formation introduced a new dimension in tailoring ammonia adsorption efficiency. The bead sample containing 20% CaCl_2_ (AC-B (20%)), with pH-8 crosslinking solution, has shown the two times more ammonia adsorption due to optimum crosslinking, physicochemical interactions and the presence of tuned amount of calcium chloride (CaCl_2_) on the surface and pores. SEM, XRD, FTIR, BET, DFT, and XPS are used to analyse the fabricated samples before and after the adsorption to thoroughly investigate the adsorption mechanism. The kinetic analysis of experimental results was conducted by applying nonlinear Pseudo 1^st^ order (PFO), Pseudo 2^nd^ order (PSO), and Elovich models, with best fit with N-PSO model confirming the highest mass transfer by the bead sample (AC-B (20%)). Lastly, the economic feasibility analysis as well as the impact of thermal treatment on recovery and sample regeneration was studied by desorbing the best samples at four different temperatures (120 °C, 140 °C, 160 °C, and 180 °C).

**Graphical Abstract:**

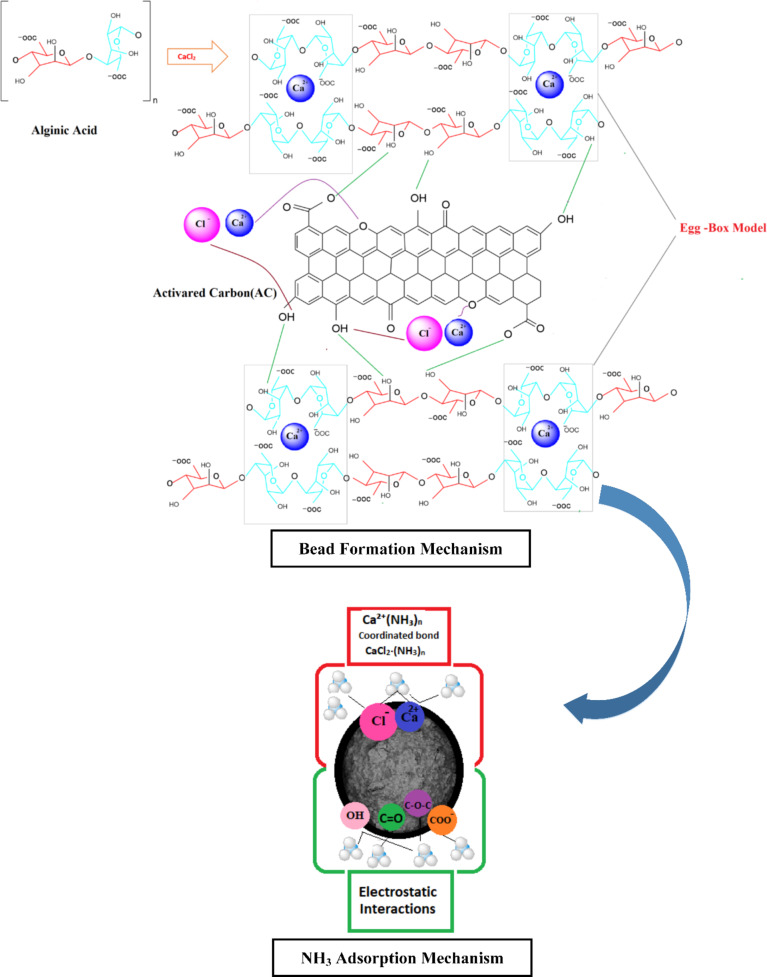

**Supplementary Information:**

The online version contains supplementary material available at 10.1007/s42247-025-01106-8.

## Introduction

Globally, the energy demand for cooling systems is increasing annually due to a number of factors, such as abrupt temperature hikes, increments in population, and economic development [1]. Recent research revealed that 14% of energy is being utilized for cooling systems in the United Kingdom, which also causes the emission of greenhouse gases [2, 3]. Moreover, the efficiency of refrigeration systems is also reduced in the daytime due to augmentation in air temperature [4]. To overcome these challenges, cold thermal energy storage (CTES) systems are receiving attention due to their flexibility in storing energy amidst low-demand times and utilizing it at peak demand [5]. However, the dependence of commercial CTES systems on traditional phase-changing materials (PCMs) is challenging owing to their poor thermal conductivity in solid phase [6]. To improve the refrigeration system, ammonia can be used as CTES medium due to its availability, lower cost (~$0.5/kg), and storage capacity (latent heat of evaporation) that varies from 1369 to 1100 kJ/kg in the temperature range (−30 to 40 °C), which is 4–6 times that of traditional PCMs (200–300 kJ/kg) [7].

Connecting with previous facts, for energy efficiency, ammonia gas as a refrigerant needs to be stored during periods of low cooling and released at high demand. For this purpose, various pristine adsorbents have been utilized for ammonia adsorption. Metal halide salts are emerging as strong candidates because of their high affinity for ammonia due to high polarity, lattice structure, and active sites that allow for strong interactions with ammonia molecules. Additionally, these materials can easily be regenerated comparatively and reused, which makes them cost-effective and environmentally friendly [8]. These qualities persuaded the researchers to analyze the ammonia adsorption using MgCl_2_, CaCl_2_, CaBr_2_, SrCl_2_, SrB_2_, NiCl_2_, and MnCl_2_ and concluded that metal salts are a good contender for ammonia adsorption [9–11]. However, there are issues of swelling and agglomeration, which can reduce the heat and mass transfer and hinder the adsorption and desorption kinetics [12, 13].

To overcome these problems, several studies have been carried out to impregnate the mesoporous materials with metal halide salts to analyze for ammonia gas adsorption. Such as V.E Sharonov et al. [8], A.M.B Furtado et al. [14], X. Pan et al. [15], and J.V. Veselovskaya et al. [13], have impregnated various matrixes (alumina, silica, MOFs, and vermiculite) with MgCl_2_, CaCl_2_, BaCl_2_, and ZnCl_2_ and concluded that the impregnation technique reduced the swelling of salts and improved the adsorption of ammonia. Other than various matrices, activated carbon is a comparably practical and environmentally sustainable material to be utilized as an adsorbent and metal halide salt carrier due to its high porosity [16–19]. Recently, J.H. Park et al. [20] loaded activated carbon with MgCl_2_ and analyzed the enhanced ammonia adsorption. Whereas, in 2023, F. Zhu et al. [21], analyzed the ammonia adsorption on activated carbon derived from hydro-char of pomelo peel, which depicted that the attached functional group can also affect the adsorbed amount of ammonia gas [22, 23]. B. J. Kim et al. [24], concluded in their research that the type of oxygen group on the surface can impact the ammonia adsorption, whereas -COOH and -OH groups can also create strong bonds with NH_3_ molecules [25, 26]. In an interesting study, C. C Huang et al. [27], pre-treated the activated carbon with nitric acid, sulfuric acid, acetic acid, phosphoric acid, and hydrochloric acid and confirmed nitric acid treatment caused more ammonia adsorption. Lately, in 2024, Z. Guozhi et al. [28], have conducted an experimental study on SO_2_ and NH_3_ adsorption by AC with monometallic active sites and concluded that Cu/AC has shown good ammonia adsorption. The use or discharge of biochar produced from biomass conversion is another important concern. The use of biochar from biomass conversion as energy storage materials may release the pressure from massive biochar produced from biomass conversion.

Aforementioned studies revealed that activated carbon-based composites can be efficacious for ammonia adsorption. However, it cannot be effectively utilized from lab to pilot scale due to the following shortcomings. There is significant loss of AC particles during the separation and regeneration process due to mechanical abrasion, attrition, or detachment of fine particles from the adsorbent, which affects the consistent adsorption performance [29–31]. Powdered AC can clump together even after one adsorption/desorption cycle, which causes a reduction in effective surface area and pore accessibility. There is also the possibility that exposure of these particles in an ammonia environment can cause a change in pore structure due to less mechanical stability. To resolve these issues, the hypothesis is to structure the AC particles by encapsulating them using a binder to form beads [30]. Previously, various MOF beads have been fabricated by using green alginate hydrogel, composed of (1–4)-linked β-d-mannuronic (M) and α-L-guluronic acid [31–35], but little work can be seen for activated carbon. However, the most imperative point raised here is related to the change in amount and mechanism of ammonia adsorption as the surface chemistry, distribution, and placement of salt change drastically during both impregnation and bead formation processes.

This study aimed to analyze the synergetic effects of pure, CaCl_2_-impregnated AC powder and AC beads on ammonia adsorption. Additionally, the study explored the impact of salt distribution in both powdered and bead samples, as well as the role of salt as a crosslinking agent in bead formation, which influenced the surface chemistry and, in turn, the ammonia adsorption process. While earlier studies highlighted the importance of a matrix (e.g., activated carbon), various salts (e.g., MgCl_2_, ZnCl_2_, and CaCl_2_), and functional groups in influencing adsorption behavior, they generally overlooked the impact of testing on powdered samples versus structured bead forms. The unique aspect of this manuscript is the examination of bead formation by controlling the pH of the dipping solution. By maintaining a pH of 8, the alginic acid is optimally deprotonated, which enables effective crosslinking with Ca²⁺ ions. This interaction facilitates the formation of a well-structured, highly porous network that maximizes the accessibility of adsorption sites for ammonia gas. The experimental results were validated using nonlinear Pseudo 1^st^ order (PFO), Pseudo 2^nd^ order (PSO), and Elovich models. Furthermore, the adsorption mechanism was substantiated through the use of XRD, FTIR, BET, DFT, and XPS techniques, and systematic desorption analysis was conducted at four different temperatures, and recyclability was carried out at 180 °C to analyze the impact of thermal treatment on recovery and sample regeneration. Last but not least, an economic analysis was also performed to compare the feasibility over costly conventional adsorbents.

## Materials and methods

### Materials

The activated carbon (AC) powder was purchased from Sigma-Aldrich (Merck), United Kingdom (UK). For impregnation, CaCl_2_ salt was obtained from Sigma-Aldrich (Merck), UK. For encapsulation, alginic acid and CaCl_2_ were sourced from Sigma-Aldrich (Merck), UK. For pH control, sodium hydroxide (NaOH) and acetic acid were purchased from Sigma-Aldrich (Merck), UK. The test gases dry nitrogen and ammonia (99.9% purity) were procured from the British Oxygen Company (BOC) - Linde Group, UK.

### Methods

#### Impregnation of AC-powder

All sample bottles and Petri dishes were thoroughly cleaned using acetone and dried in an oven at 60 °C. The required quantity of activated carbon (AC) powder was dried at 80 °C for 24 h in an oven. To prepare the solution, the optimized quantities of CaCl_2_ salts were dissolved in distilled water and continuously stirred for 2 h. Subsequently, the AC particles were added to the salt solution and left to stir for 24 h. The mixture was then filtered using a vacuum filtration setup, and the composite material was collected on filter paper. The filtrate was heated for 24 h at a temperature ranging from 60 °C to 80 °C and collected in sample bottles to analyze the salts remaining in solutions. This process was repeated for the following salt percentages, ranging from 0 wt% to 30 wt%, using the same method as described above. The nomenclature used for these samples is AC-P, AC-P (10%), AC-P (20%), and AC-P (30%).

### AC bead fabrication

To manufacture the beads of activated carbon, 5 g of alginic acid is dissolved in distilled water and stirred for 12 h, then the required amount of AC particles is mixed with the alginic acid solution and stirred for 12 h. Three percentages, such as 10 wt%, 20 wt%, and 30 wt%, of CaCl_2_ were dissolved in 20 ml of distilled water (separately) and stirred for 30 min. Afterwards, the AC-alginate acid solution dropped in salt solutions using a syringe, which crosslinked in AC beads. These beads were kept in salt solution for 2 h and then washed five (5) times with distilled water. In the end, the beads were freeze-dried for 20 h and kept airtight in sample bottles. The nomenclature used for the samples is given as AC-B (10%), AC-B (20%), and AC-B (30%).

### AC bead fabrication at various pH of crosslinking salt solution

Initially, the procedure of AC bead formation is the same as mentioned above. CaCl_2_ solution was synthesized by dissolving 20 wt% of salt in distilled water. Afterward, CaCl_2_ solution was prepared by dissolving 20 wt% of CaCl_2_ in distilled water. The pH of the solution was adjusted using 0.2 M acetic acid/0.1 M NaOH solutions. The desired pH was achieved by gradually adding acetic acid or NaOH to the CaCl_2_ solution while continuously monitoring the pH using a pH meter during the mixing process. Subsequently, the AC-alginic acid solution was added dropwise into the CaCl_2_ solution to form beads. These beads were kept in salt solution for 2 h and washed with distilled water five (5) times. In the end, the beads were freeze-dried for 20 h and kept airtight in sample bottles. The nomenclature used for the sample is given as AC-B (pH-4), AC-B (pH-6), AC-B (pH-8), AC-B (pH-9), AC-B (pH-10), and AC-B (pH-12).

### Characterization of composition and structure

The surface morphology and dimensional analysis of fabricated samples were carried out by Carl Zeiss SIGMA HD VP FEG SEM analytical low vacuum SEM, which was provided by JEOL Ltd., Japan. The analysis was performed at two different accelerating voltages, namely 15 kV and 20 kV. The crystallization analysis of samples was carried out using the Bruker D-2 Phaser X-ray diffractometer, which is equipped with a copper sample holder and utilizes Cu-Kα radiation with a wavelength of 1.5406 Å. The instrument was operated at a voltage of 30 kV and a current of 10 mA to perform the X-ray diffraction (XRD) analysis. An FTIR analysis was conducted over a wide range of wave numbers, spanning from 400 cm^−1^ to 4000 cm^−1^, with a high resolution of 4 cm^−1^. In this technique, a small quantity of finely ground sample was mixed with dry potassium bromide (KBr) powder, and the mixture was compressed under high pressure to form a transparent pellet. This pellet was then analyzed using a Shimadzu IRTracer-100 FTIR spectrometer made by Harrick Scientific, allowing for detailed characterization of molecular vibrations and functional groups present in the samples. To examine the chemical composition and observe variations in binding energy before and after ammonia adsorption, the selected samples were characterized using X-ray photoelectron spectroscopy (XPS). The analysis was performed using a Thermo Scientific NEXSA G2 system, equipped with a monochromated Al Kα X-ray source (1486.7 eV), a spherical sector analyzer, and 128-channel detectors.

### Pore structure analysis

The analysis of specific area and pore volume was conducted using the Quanta Chrome Autosorb IQ gas adsorption analyzer. Using this physisorption equipment, the BET surface area, pore volume, and average pore diameter were determined. Fabricated samples (pure, impregnated powder, and beads) were degassed at 100 °C for 12 h before mounting in the analyzer. The analysis of data was carried out by Quantachrome^®^ ASiQwin software. The surface area was calculated using the Brunauer-Emmett-Teller (BET) equation, whereas pore volume and average pore diameter (slit-like structure) were estimated using Eq. (1) and Eq. (2). Pore volume was also determined using the DFT method (slit pore, NLDFT equilibrium model). The characteristic adsorption energy was calculated using the Dubinin–Radushkevich Eq. (3), as applied in previous research work [36–38]:

1 $$\:{V}_{t}=\frac{{Q}_{0.99}}{647}$$

2 $$\:L_0=\frac{2\times\:V_t}{S_{BET}}\times1000$$

3 $$E_o=\left(10.8/L_o\right)+11.4$$

Where *V*_*t*_ is the total pore volume (cm^3^/g), *Q*_*0.99*_ is the gas adsorbed in volume at relative pressure 0.99, L_1_ represents the average pore diameter (slit-like structure) in nm, *S*_*BET*_ is the BET surface area (m^2^/g), and *E*_*o*_ is the characteristic adsorption energy (kJ/mol) [36–38].

### Ammonia adsorption analysis

**Fig. 1:**
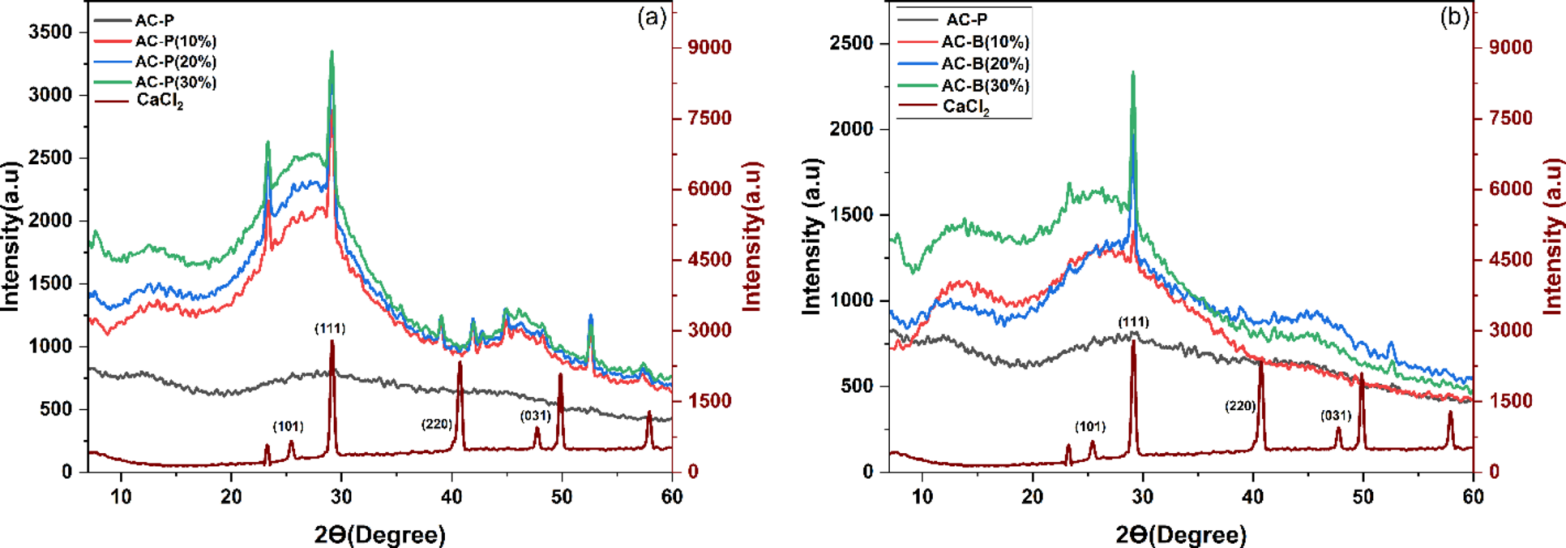
(**a**) XRD curves of AC-Powdered samples (**b**) XRD curves of AC-Bead samples

The dynamic ammonia adsorption analysis of samples is conducted at room temperature and atmospheric pressure in the designed rig, as shown in Fig. 1S. The system is composed of dry nitrogen and ammonia cylinders. The flow rates of both gases are controlled by mass flow controllers using Flow View software. The whole rig is purged with nitrogen gas before and after placing a 1 g sample inside the glass column. The ammonia gas is introduced into the system and recorded the flow through the first mass flow meter, controlled by LabView software. Subsequently, after achieving static flow, it traversed through a packed glass column (measuring 6 cm in length and 20 mm in outer diameter) and the second mass flow meter. The concentration of the eluent gas was determined by mass spectrometry (QMG 250 PRISMAPRO) and analyzed using PVMassSpec software. The outlet and inlet concentration of ammonia gas were compared and indicated breakthrough at (C/C_o_=1). The regeneration studies of the sample are conducted at various temperatures (120 °C, 140 °C, 160 °C, and 180 °C).

The dynamic adsorption and desorption capacity are calculated using the following equations. 

4 $$\:V=\frac{{\int\:}_0^tvdt-V_{max/min}}W$$

5 $$\:n=\frac{PV}{RT}$$

Where $\:v\:$ is the flow rate of ammonia gas in ml/min. *V*_*max/min*_ is the maximum (adsorption) and minimum (desorption) flow rate in ml/min. *W* is the mass of the sample inside the glass column (g), *t* is the retention time (s), *P* is pressure (kPa), *R* is the gas constant, and *T* is the measurement temperature [39].

### Kinetic model analysis

To evaluate the mechanism involved in ammonia adsorption inside powdered and bead samples, three models, such as non-linear Pseudo-first order (N-PFO), non-linear Pseudo-second order (N-PSO), and non-linear Elovich (N-E) models, have been applied [40].

The N-PFO model assumes that the kinetic adsorption rate is proportional to the number of sites available on adsorbents. The interaction between adsorbent and adsorbate is more pronounced physically. The Eq. (6) is the non-linear pseudo-first-order Eq. 

6 $$\:{q}_{t}={{q}_{e}\left(1-{e}^{-k1t}\right)}_{\:}$$

The N-PSO model assumes that the adsorption rate is proportional to the square of the number of available sites and also represents the nature of interactions between adsorbate and adsorbent as chemical. The equation can be written as:

7 $$\:q_t=\frac{q_e^{2\:}k_2\:t}{1+q_ek_2t}$$

Where *t* is the time (s) of adsorption and *q*_*e*_ and *q*_*t*_ (mg/g) are the amounts of ammonia adsorbed on the adsorbents at equilibrium and at any time. Whereas the equilibrium rate constants of the first and second-order pseudo equations are *k*_*1*_ (g/mg.s) and *k*_*2*_ (s^−1^) [41, 42].

The non-linear Elovich model validates the adsorption on heterogeneous surfaces, and interaction is more chemical in nature. The equation is given below:

8 $$\:q_t=\frac1\beta\:\ln(1+\alpha\:\beta\:t)$$

Where *q*_*t*_ (mg/g) is the amount of adsorbed ammonia at time t, *β* is the desorption constant (g/mg), *α* is the initial adsorption rate (mg/g.s), and *t* is the time (s).

## Result and discussion

### Composition and morphology analysis

The qualitative and quantitative analyses of fabricated samples were conducted by using SEM, XRD, FTIR, BET, DFT, and XPS techniques, as explained below.

Figure 1 (a) and (b) depict the comparative reflections in X-ray diffraction curves of AC composite powdered and bead samples. The CaCl_2_ peaks are visible at 23.2°, 25.6°, 29.2°, 40.7°, and 47.6° [43], which represent its crystalline nature. On the contrary, the AC is amorphous, so broad peaks around 26° and 43° can be observed [44]. In the case of AC powder impregnated with CaCl_2_, the prominent peaks highlight the presence of calcium chloride in pores, and the intensity of Bragg peaks increased as the concentration of calcium chloride increased from 10 to 30 wt%. On the contrary, the beads are fabricated by dropping an AC-alginic acid mixture in the calcium chloride solution, and calcium ions crosslink the polymeric chains to form solid beads. The presence of salt is confirmed by the presence of main peaks at 23.2° and 29.2°. Secondly, the intensity of peaks increased with salt concentration, which concluded that salt residence inside the bead and on the surface augmented as concentration increased from 10 to 30 wt%.

**Fig. 2:**
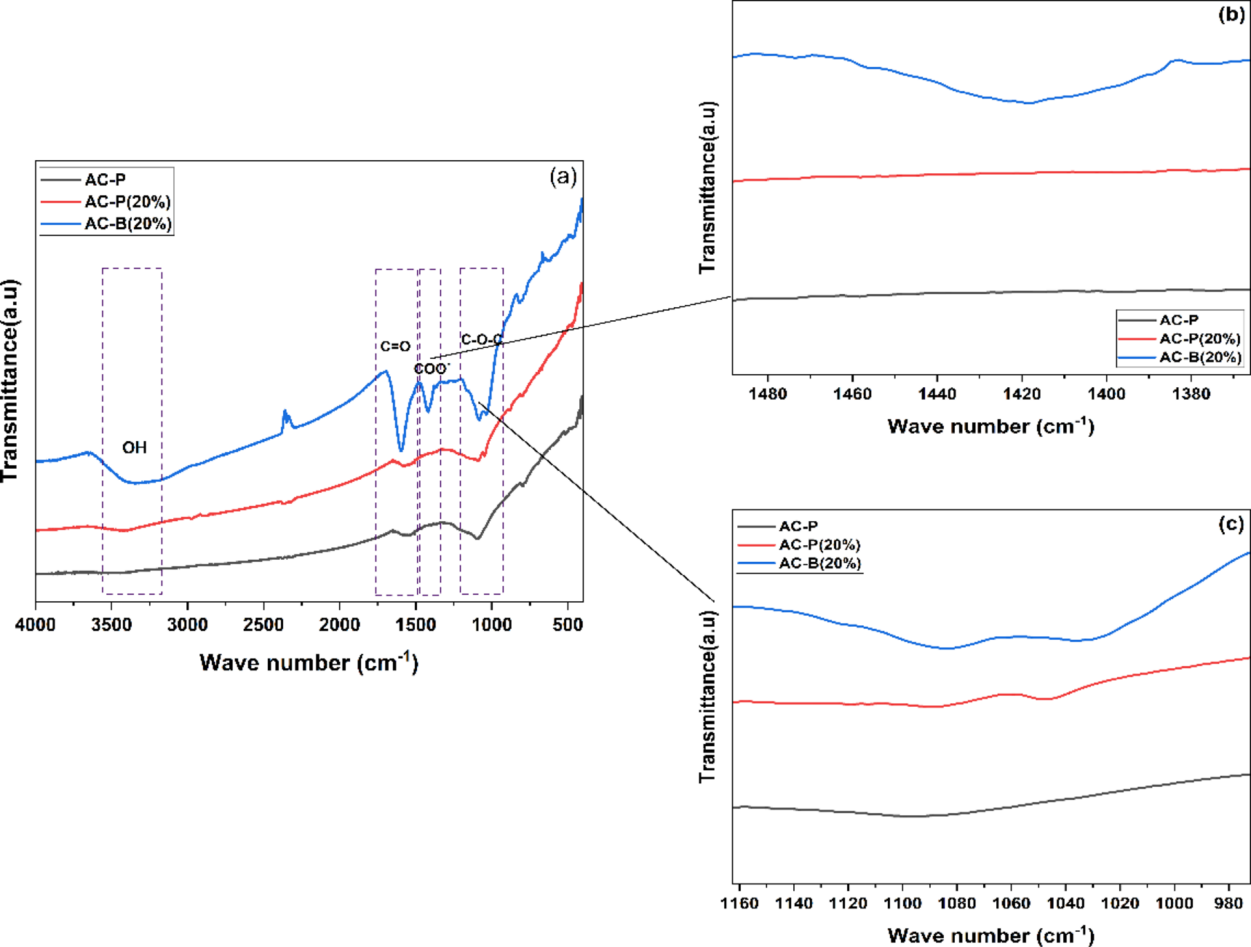
(**a**) FTIR spectrum of AC-P, AC-P (20% and AC-B (20%) samples, (**b**) magnified spectrum of COO^-^ peak, and (**c**) magnified spectrum of C-O-C peak

The FTIR analysis of the fabricated samples in Fig. 2 provides us with imperative information about the presence of functional groups and various interactions. In the case of pure activated carbon, the peak intensities are low. The peak range of 3000–3500 cm⁻¹ represents the stretching vibration of O-H in carboxyl and hydroxyl groups, which are inherent functional groups of activated carbon and probably due to incomplete carbonization, oxidation, or chemisorbed water [45, 46]. The observed –OH contributions can be attributed to alginate, activated carbon, and moisture in CaCl_2_ [47–50]. Additionally, a minor peak near 2400 cm^−1^ was observed and is attributed to background CO_2_ interference. Despite rigorous sample preparation including 24 h drying of samples and KBr in oven and rapid sample transfer, this CO_2_ contribution is inevitable, consistent with literature reports [47, 51–53]. Another broad peak at 1500–1650 cm⁻¹ depicts the presence of C=O stretching vibrations due to carbonyls, and carboxylic acids. Whereas, the peak at 1095 cm⁻¹ highlights the vibrations of C-O stretching due to carboxylic and phenolic groups, which is in accordance with the previous research [47, 54]. Likewise, as the AC-P (20 wt%) was fabricated by impregnating CaCl_2_ salt, the FTIR curve of AC-P (20 wt%) shows similar peaks. However, the peak in the range of 1040 cm⁻¹ to 1087 cm⁻¹ split and shifted towards the low-frequency range. This might be due to the electrostatic interaction between Ca^2+^ ions and the C-O-C bond, as the positively charged metal ions interact with the oxygen atom containing the partial negative charges. These interactions modify the vibrational frequencies of bonds, resulting in peak change [55, 56].

**Fig. 3:**
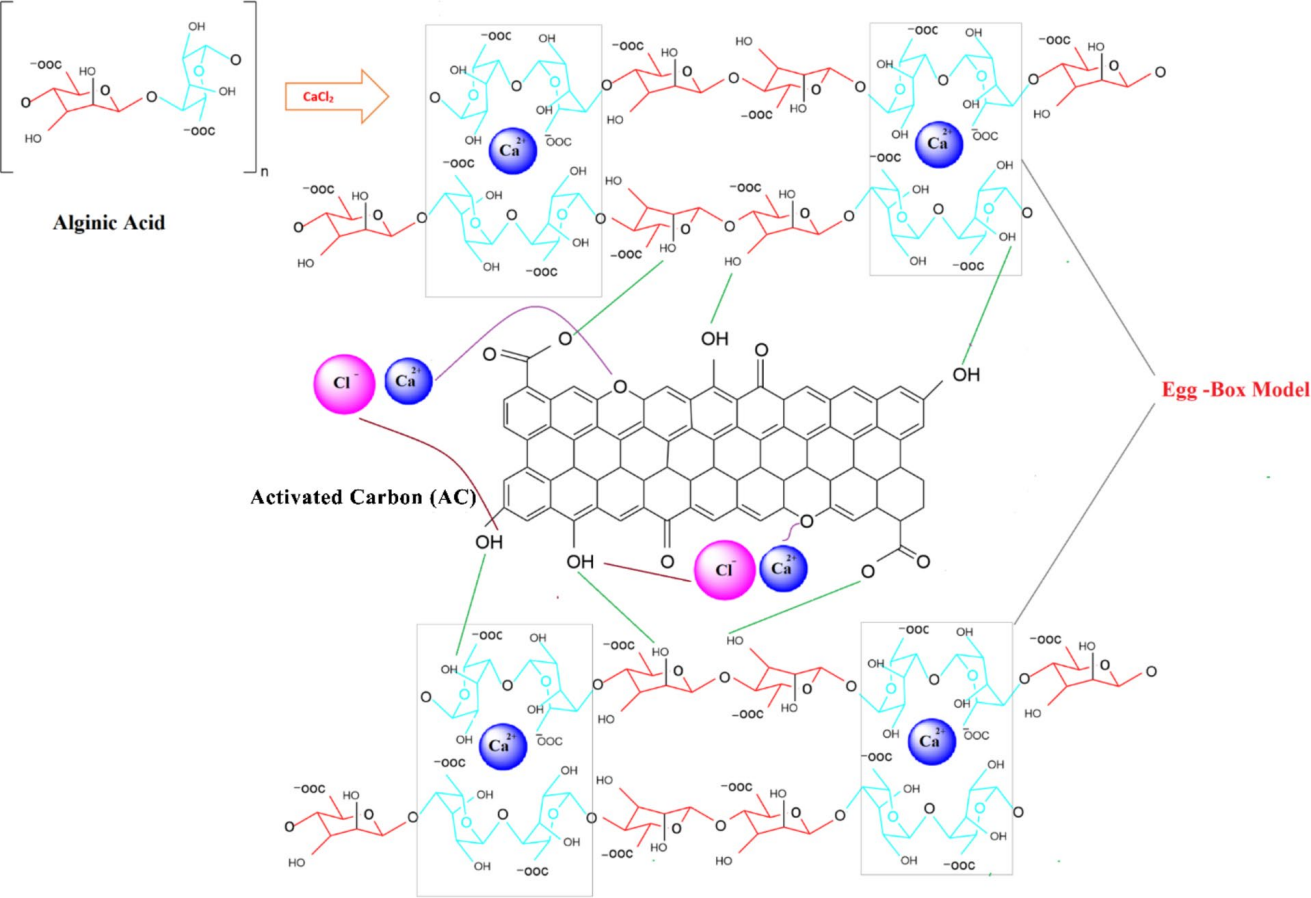
A schematic representation of bead formation mechanism

On the contrary, the peaks in the AC-B (20 wt%) sample are more intense. As previously mentioned, the AC-B (20 wt%) bead sample contains activated carbon, crosslinking calcium chloride salt, and binder alginic acid containing guluronic and mannuronic acid. Many researchers have proven that alginate always followed the ordered sequence through dimerization of guluronic monomer in the presence of Ca^2+^ ions. The buckled shape of poly-guluronic sections aligned the two chain sections, contributing to the creation of coordination sites with cavities favourable for divalent cations such as Ca^2+^ [57–59], interconnected with carboxylate and other oxygen atoms to form the Egg-Box Model [58–61]. So according to the FTIR spectrum, the broad peak at 3300 cm⁻¹ depicts the presence of the O-H group, and the peak at 1600 cm⁻¹ is representative of the C=O group. However, a new peak, observed at 1417 cm⁻¹, corresponds to the symmetric stretching peak of carboxylate salt groups (-COO⁻ groups), due to guluronic and mannuronic monomers in alginic acid, which further interact with Ca²^+^ ions [51, 62]. The shifting and splitting of peak 1080 cm^−1^ is also depicting calcium chloride’s interaction with oxygen-containing functional groups on alginic acid and activated carbon. Probably, when Ca^2+^ ions interact with multiple oxygen atoms from different functional groups that creates an environment in which the oxygen atom acts as a bridge between two carbon atoms. This corroborates the presence of salt in beads [60, 63]. The schematic representation of the bead mechanism is given in Fig. 3.

Following the FTIR analysis, the TGA results further validate our interpretations. As shown in Fig. 2S, the thermogravimetric analysis (TGA) of both AC-B(10%) and AC-B(20%) samples reveals two distinct events. An initial weight loss between approximately 50 °C and 180 °C can be attributed to the evaporation of moisture and the loss of volatile compounds from alginic acid, activated carbon, and CaCl_2_. Subsequently, a second weight loss event occurring after 225 °C corresponds to the decomposition of alginic acid chains, thereby confirming its presence in the samples. These observations are consistent with previous studies [64–68] and reinforce our conclusions regarding the material composition.

**Fig. 4:**
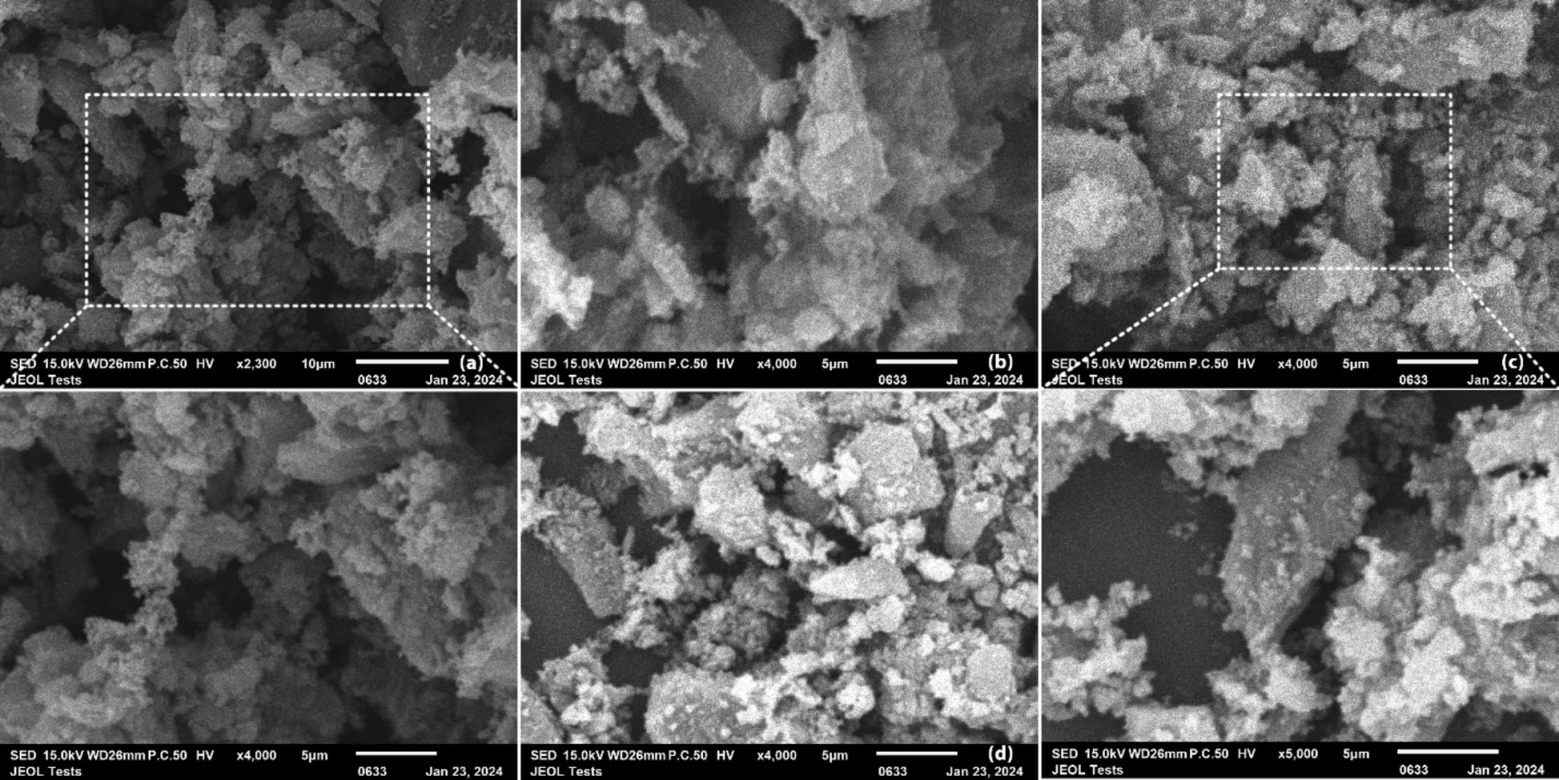
SEM image of (**a**) AC-P, (**b**) AC-P (10%), (**c**) AC-P (20%), and (**d**) AC-P (30%)

The particle morphology was analyzed by using the scanning electron microscopy technique. According to the analysis, the amorphous structure of activated carbon powder is visible in Fig. 4. The aggregation of adsorbents can be observed more with the addition of CaCl_2_ from 10 to 30 wt% [69]. The magnified image can show a better view of the AC-P (20 wt%) sample.

**Fig. 5:**
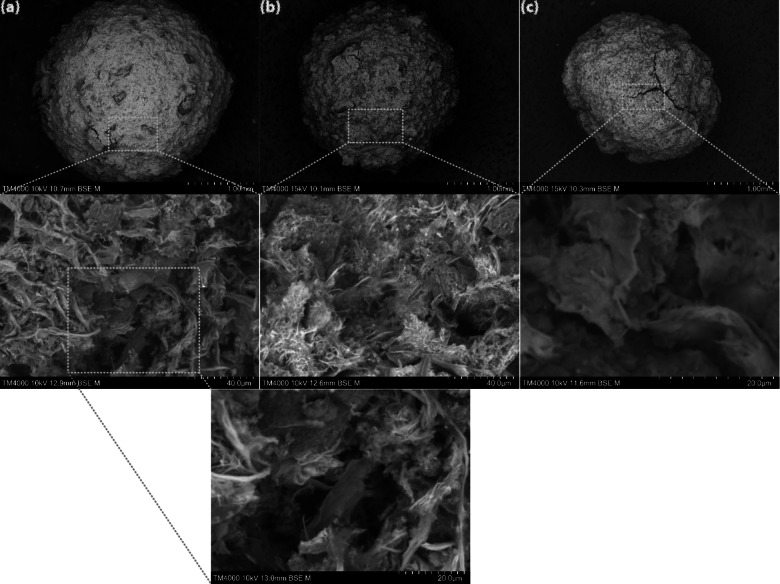
(**a**) SEM image of AC-B (10%), (**b**) AC-B (20%), and (**c**) AC-P (30%)

The SEM analysis of AC beads is depicted in Fig. 5 (a), (b), (c). As per analysis, all of the fabricated beads were porous and comprised of cracks on the surface as the concentration of salt augmented in the dipping solution, specifically with 30% salt. In the case of AC-B-10% and AC-B-20%, at high magnification, the gel-like network represents polymeric fibers, chemically engaged with AC particles. This observation aligned well with previous studies, such as Ayarza et al. [70], which explained the impact of alginic acid gelation on the morphology of beads. As the alginic acid and AC solution were introduced in the CaCl_2_ aqueous solution, the cation Ca^2+^ interacted with the anionic polyelectrolyte on the interface, resulting in the formation of an outer shell. As Ca^2+^ ions diffused inside, it completely solidified and converted into beads [71–73]. This interconnection is due to the interaction of calcium ions with oxygen atoms of guluronic monomers and activated carbon, supported by FTIR analysis.

### Analysis of pore structure

Nitrogen sorption was conducted for all fabricated samples to analyze the pore structure and salt contents. As shown in Table 1, the pore volume of pure AC powder is 0.689 cm^3^/g, and BET surface area (S_Bet_) is 666 m²/g. However, with the addition of salt from 10 wt% to 30 wt%, the pore volume reduced from 0.78 (AC-P-10 wt%), 0.61 (AC-P-20 wt%) and 0.53 (AC-P-30 wt%). Similarly, the BET surface area also reduced from 573 m²/g (10 wt%), 444 m²/g (20 wt%), to 385 m²/g (30 wt%). The reduction in surface area and pore volume shows that the salt is embedded inside the pores of AC powder. In the case of AC beads, by increasing the salt content in the dipping solution, BET surface area (S_Bet_) reduced from 436 m²/g for AC-B-10%, 421 m²/g for AC-B-20%, and 300 m²/g for AC-B-30%. The pore volume reduced from 0.55 cm³/g for AC-B-10%, 0.33 cm³/g for AC-B-30% but 0.57 cm³/g was observed for AC-B-20%, comparatively.

**Table 1:** BET specific surface area (S_Bet_), pore volume, average pore diameter (Slit) and characteristic adsorption energy

Samples	S_Bet_	Total Pore volume (V_t_)	Avg. pore diameter (L_0_) (slit)	Characteristic adsorption energy (E_o_)
	(m^2^/g)	(cm^3^/g)	(nm)	kJ/mol
AC-P	666	0.81	2.43	15.92
AC-P (10%)	573	0.78	2.72	15.45
AC-P (20%)	444	0.61	2.74	15.42
AC-P (30%)	385	0.53	2.75	15.40
AC-B (10%)	436	0.55	2.52	15.76
AC-B (20%)	421	0.57	2.70	15.47
AC-B (30%)	300	0.33	2.20	16.38

Connecting previous characterization results with BET results revealed a very significant finding that two phenomena occurred by dropping the alginic acid-activated carbon solution in calcium chloride salt solution.

1. The calcium ion crosslinked the guluronic chains in the alginic acid binder, forming the egg-box model, while binder chains also interacted with activated carbon, confirmed by FTIR and SEM analysis [61].
2. The remaining amount of CaCl_2_ got embedded inside the pores of activated carbon, as alginic acid chains only interacted with activated carbon particles, unable to block pores [34].

**Fig. 6:**
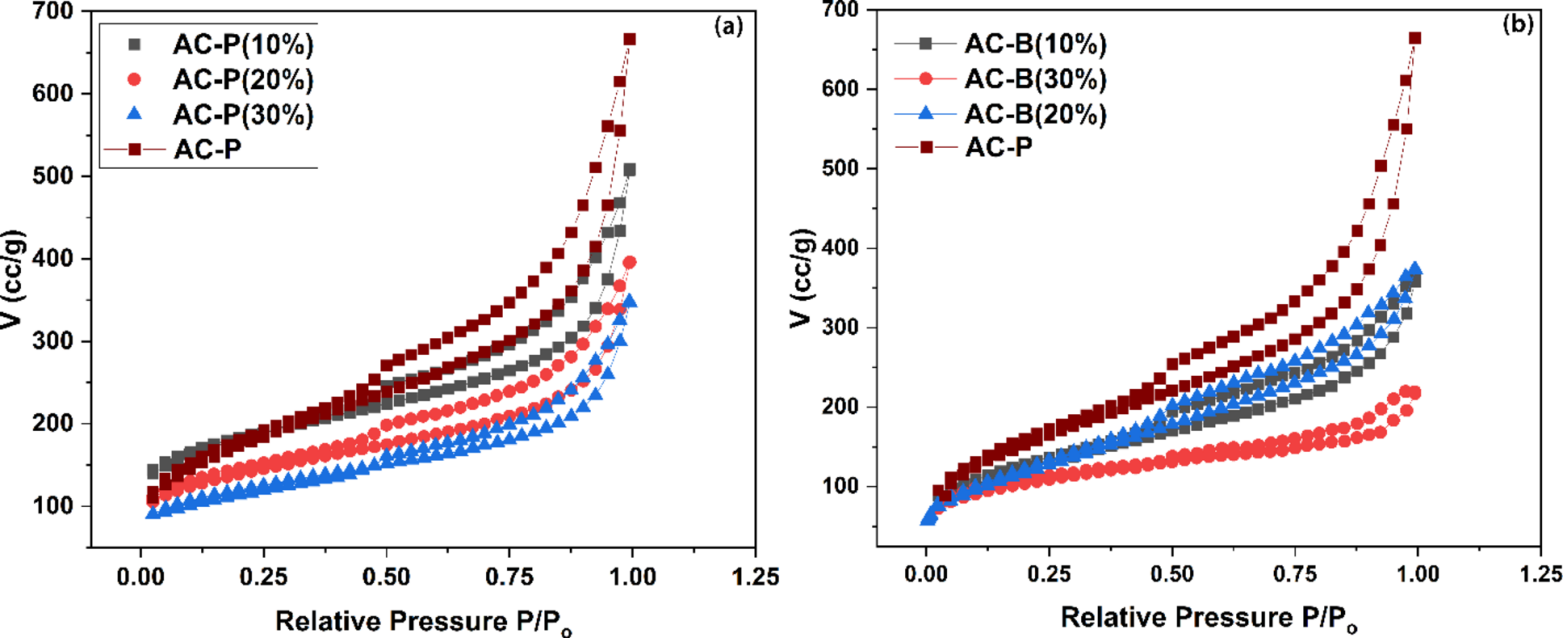
N_2_ adsorption and desorption isotherms for (**a**) powder and (**b**) bead samples, derived from N_2_ isotherms at −196 °C using the BET method

The salt impregnation is affected by various factors such as pore size and pore volume, pore structure, connectivity between pores, and surface chemical interactions. Here, further analyzing the BET data has provided information on types of pores and how they are impacted by salt impregnation. According to Fig. 6; Table 1, the adsorption curves for pure and composite samples are the combination of type I and type IV isotherms in IUPAC classification, and adsorption-desorption isotherms represent a loop when P/P_o_ > 0.4. This behavior indicates the presence of more mesopores as compared to micropores in the samples. The rationale is that during adsorption, the gas condenses inside the mesopores at higher relative pressures, and in the course of desorption, the gas requires more time to desorb due to capillary condensation [74, 75]. This delay causes the generation of different hysteresis loops, which furnish information about the type or shape of pores [76]. Here, the hysteresis loop of AC-P and AC-P (10 wt% - 30 wt%) depicts a type H3 hysteresis loop, confirming the presence of slit-like pores. The computed results of average pore diameter revealed an increasing trend for salt-impregnated samples, which might be due to the filling of small pores with salt, shifting the average pore diameter to large pores [77–79]. Furthermore, according to the DFT analysis data as represented in Fig. 3S (a) and Table 1S, the pure sample of AC-P has more mesopore volume (0.495 cm^3^/g) as compared to the micropore volume (0.194 cm^3^/g). However, as the amount of impregnated salt increased from 10 wt% to 30 wt%, the pore volume decreased, comparably.

In the case of AC beads, the adsorption curve is still the combination of type I and type IV isotherms in IUPAC classification; however, a little shift in the hysteresis loop was observed. As the salt concentration in the dipping solution increased, the hysteresis loop became a combination of H3 and H4 types. This combination highlights the presence of complex pore structure, as the beads have activated carbon powder, gelled with alginic acid in the presence of CaCl_2_, which caused diverse porosity due to AC-P (slit-like pores) and the bead itself. Similarly, as per the BET analysis data in Table 1, the increasing trend for the average pore diameter of beads was observed, with a high value for the AC-B-20% sample [77–86]. Moreover, according to the DFT data as shown in Table 1S and Fig. 3S (b), the AC beads exhibit lower microporous volume and higher mesoporosity compared to the powdered sample. The porosity trends are complex due to the dual role of CaCl_2_, which both crosslinks the alginic acid and penetrates the pore structure. By increasing the salt concentration from 10 wt% to 20 wt% resulted in a decreased in micropore volume (0.104 cm³/g, 0.086 cm³/g) and an increased in mesopore volume (0.405 cm³/g, 0.434 cm³/g). This shift likely enhanced the diffusion of ammonia gas from the surface into the pores, potentially increasing ammonia adsorption. However, at 30 wt% salt impregnation (AC-B), both micropore volume (0.048 cm³/g) and mesopore volume (0.262 cm³/g) were reduced, which may adversely affect gas diffusion and overall adsorption capacity.

### Ammonia adsorption and kinetic analysis

**Fig. 7:**
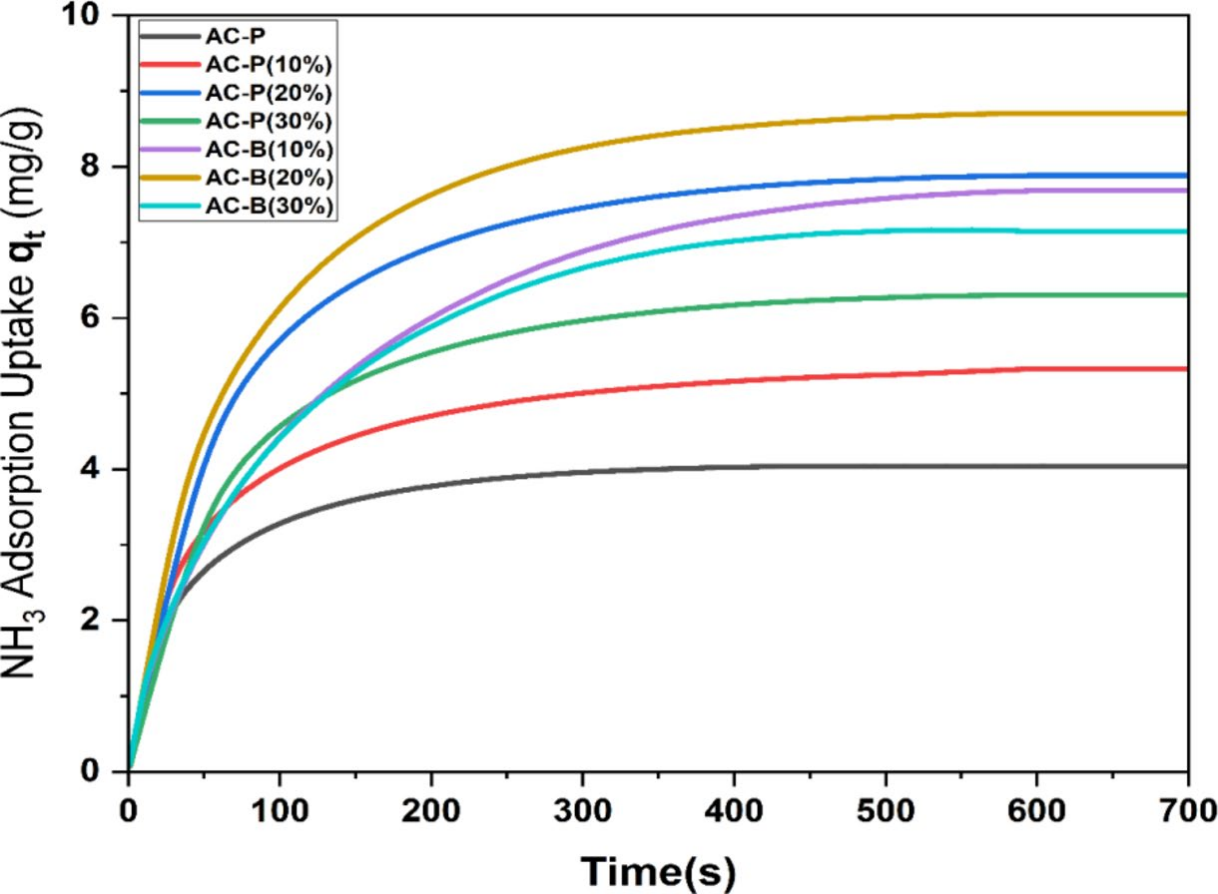
Ammonia adsorption curves of AC-P (10%, 20%, and 30%) and AC-B (10%, 20%, and 30%) samples at standard temperature and pressure

The comparative analysis of the ammonia adsorption using AC powder and AC beads revealed interesting facts. According to Fig. 7, the adsorption capacity of pure AC powder is low and nearly 4.04 mg/g. However, the ammonia adsorption increased with an increment in the concentration of calcium chloride salt inside the AC pores. This is complemented by the previously published data that calcium chloride has a loving affinity with ammonia gas [8, 87]. However, notably, the adsorption capacity of the AC-P (20%) sample is significantly more, 7.88 mg/g, as compared to 5.33 mg/g for AC-P (10%) and 6.30 mg/g for AC-P (30%). The increase in the salt concentration from 20 wt% to 30 wt% (during impregnation) resulted in the blockage of pores, causing a decrement in ammonia adsorption. These results are in accordance with the BET and DFT analysis results.

Similarly, in the case of AC beads, the ammonia adsorption augmented with an increment in the concentration of calcium chloride in the dipping solution from 10 wt% (7.68 mg/g) to 20 wt% (8.70 mg/g). Conversely, 30 wt% of salt resulted in hard bead structure and blockage of pores. This caused a reduction in ammonia adsorption to the value of 7.14 mg/g, relatively less than the AC-B-(10%) sample. The comparative analysis revealed that AC bead with 20 wt% salt has adsorbed 2 times more ammonia than the AC powdered sample.

The numeric adsorption discussion focuses on the following points.

1. Traversing ammonia gas from the external surface of pure and salt-impregnated powdered adsorbents to the pores. The impregnation of salt caused significant mass transfer.
2. Transport of ammonia gas from the external surface of bead adsorbents diffuses through porous particles (linked by binder) and is adsorbed on the internal surfaces of porous material.
3. Adsorption of ammonia depending upon the chemistry and the functional groups of adsorbents leads to significant mass transfer.

**Fig. 8:**
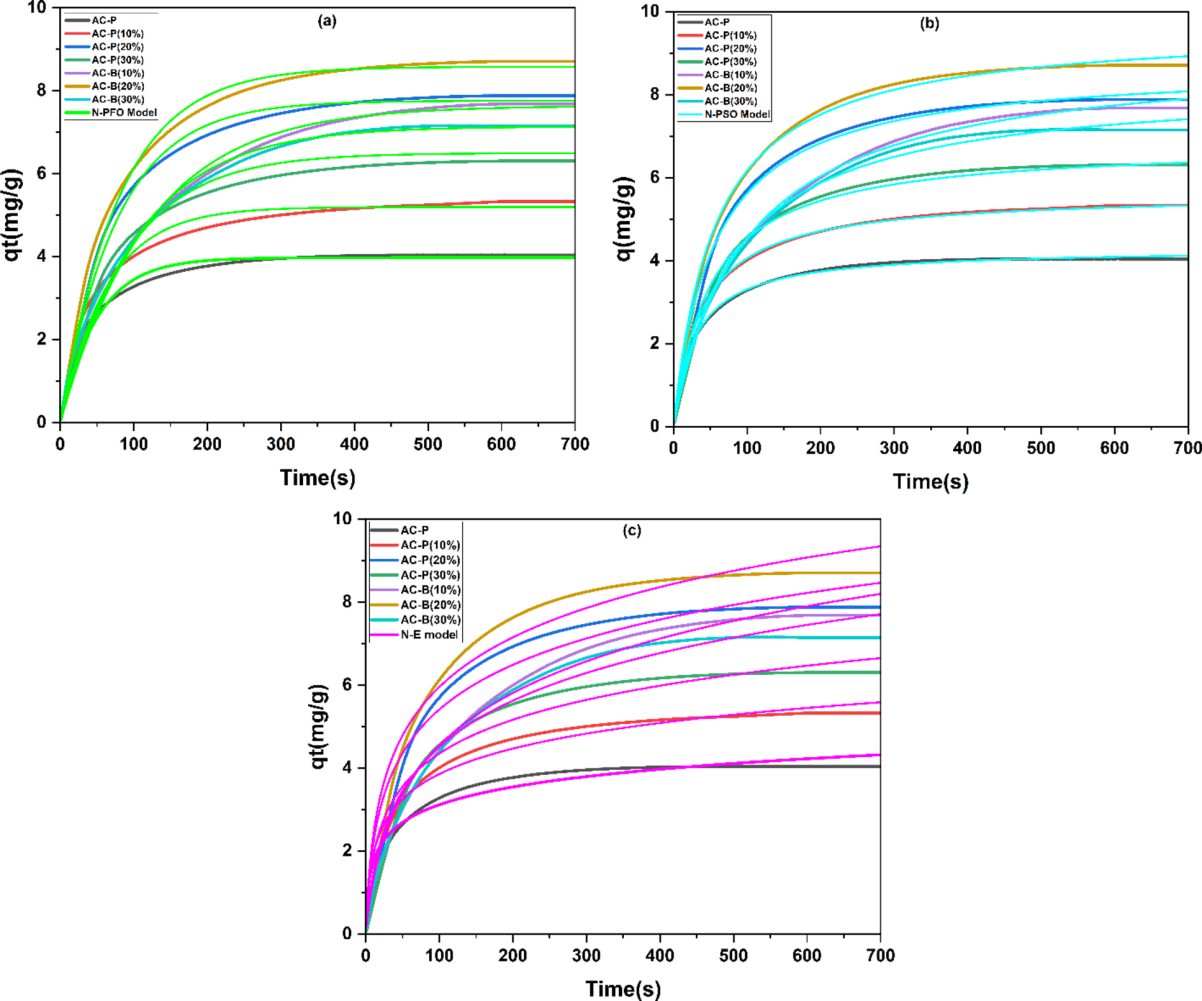
(**a**) Non-linear Pseudo 1 st Order, (**b**) Non-linear Pseudo 2nd Order, and (**c**) Non-Linear Elovich model analysis

This states that the adsorption of ammonia on pure AC, impregnated AC, and AC beads with salt is a two-step process. Initially, the external mass transfer occurred, as the ammonia gas attracted towards the surface of adsorbents and then adsorbed at active sites. The first step is important and highlights the significance of the adsorbent’s surface chemistry. To investigate the thorough mechanism, non-linear pseudo-first-order (N-PFO), non-linear pseudo-second-order (N-PSO), and non-linear Elovich (N-E) models were applied to experimental data. The rate constants (k_1_, k_2_, α, and β) and correlation factor (R²) are calculated using the Origin software. The curves and tabulated data are given below in Fig. 8; Table 2.

**Table 2:** Constants of Non-linear Pseudo 1^st^ order, Non-linear Pseudo 2^nd^ order and Non-Linear Elovich model

Samples	*N*-PFO Model		*N*-PSO Model		*N*-E model		
	k_1_ (s^−1^)	*R*^2^	k_2_ (g/mg.s)	*R*^2^	α(mg/g.s)	β(g/mg)	*R*^2^
AC-P	0.011	0.91	0.077	0.990	0.17	1.60	0.87
AC-P (10%)	0.013	0.86	0.045	0.992	0.46	1.11	0.84
AC-P (20%)	0.015	0.88	0.020	0.984	0.66	0.62	0.81
AC-P (30%)	0.012	0.83	0.029	0.992	0.44	0.83	0.80
AC-B (10%)	0.015	0.86	0.013	0.983	0.51	0.56	0.79
AC-B (20%)	0.017	0.88	0.011	0.995	0.73	0.50	0.81
AC-B (30%)	0.013	0.85	0.014	0.987	0.48	0.58	0.80

As per Fig. 8(a) and Table 2, the non-linear pseudo-first-order model (N-PFO) did not fit very well on the experimental data as the value of the correlation factor R^2^ is in the range of 0.83 to 0.91. However, the value of reaction constant k_1_ is low for pure AC powder and high for the AC-B (20%) sample, which confirms the adsorption rate is fast in bead samples as compared to pure AC and impregnated powdered samples due to the presence of more adsorption sites.

On the contrary, the non-linear second-order model (N-PSO) fits very well with the experimental data of all fabricated samples, as the R^2^ value is in the range of 0.983 to 0.995, and the value of k_2_ is low for the AC bead sample as compared to pure AC powdered and impregnated samples, which shows that the mass transfer rate is higher in bead samples, specifically in AC-B (20%), due to additional adsorption sites. Both of the models guide about the type of interactions between ammonia and fabricated adsorbents. As the N-PSO model fits more than the N-PFO model, which depicts that the ammonia-salt adsorption involves the dominance of chemical and multisite interactions other than physical interactions [88, 89]. To analyze further, the N-Elovich (N-E) model has been applied to experimental data, which does not fit well, as the correlation factor’s value ranges from 0.79 to 0.87. The values of the Elovich constants α and β are in agreement with the results of the N-PSO and N-PFO models. The desorption constant β is low, and the initial adsorption rate constant α is high for AC-B (20%) as compared to pure AC and the impregnated powdered sample. This provides an insight that the bead adsorbent AC-B-(20%) has more adsorption sites, than powdered sample AC-P(20%) and ammonia has more affinity to get adsorbed, confirmed by XPS analysis.

### Impact of surface chemistry

The above-discussed three kinetic models depicted that the mechanism involved in ammonia adsorption on powdered and bead samples is physicochemical. In the case of the AC-P (pure AC powder) sample, the porous structure supported the diffusion of ammonia gas, and adsorption is due to Van der Waals forces [90, 91]. However, the surface functional groups, such as hydroxyl and carbonyl groups, also played their role. Comparably, for the salt-impregnated samples (AC-P (10%, 20%, 30%)), other than Van der Waals forces, following interactions are also imperative. When ammonia gas interacts with CaCl_2_, a solid-gas interaction takes place which resulted in the formation of calcium chloride ammonia complex. The ammonia molecule donates electron to the calcium ion, which depicts the calcium ion acts as Lewis acid, accepting electron pair and the ammonia molecule as a Lewis base, donating electron pair [92, 93]. In a nutshell, when ammonia gas comes near calcium chloride, following important interactions take place, which are represented in the form of equations, given below [94, 95]:

9 $${Ca}^{2+}+{\left(NH_3\right)}_n\rightarrow{Ca}^{2+}{\left(NH_3\right)}_n$$

10 $$\:Ca^{2+}{\left(NH_{3}\right)}_n+2Cl^-\rightarrow CaCl_2{\left(NH_3\right)}_n$$

**Fig. 9:**
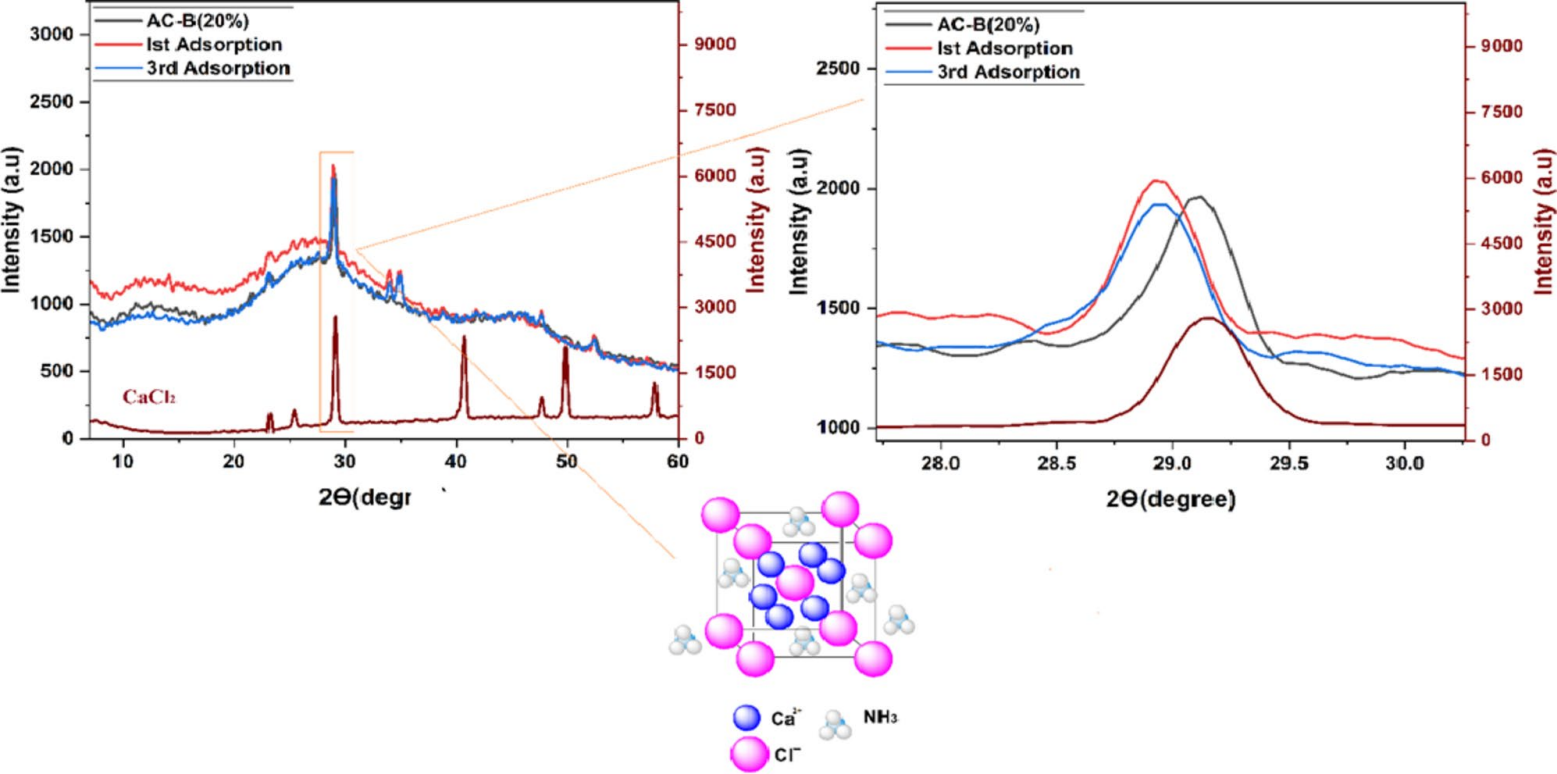
XRD analysis of AC-B (20%) sample before, after 1^st^ and 3^rd^ adsorption cycle

**Fig. 10:**
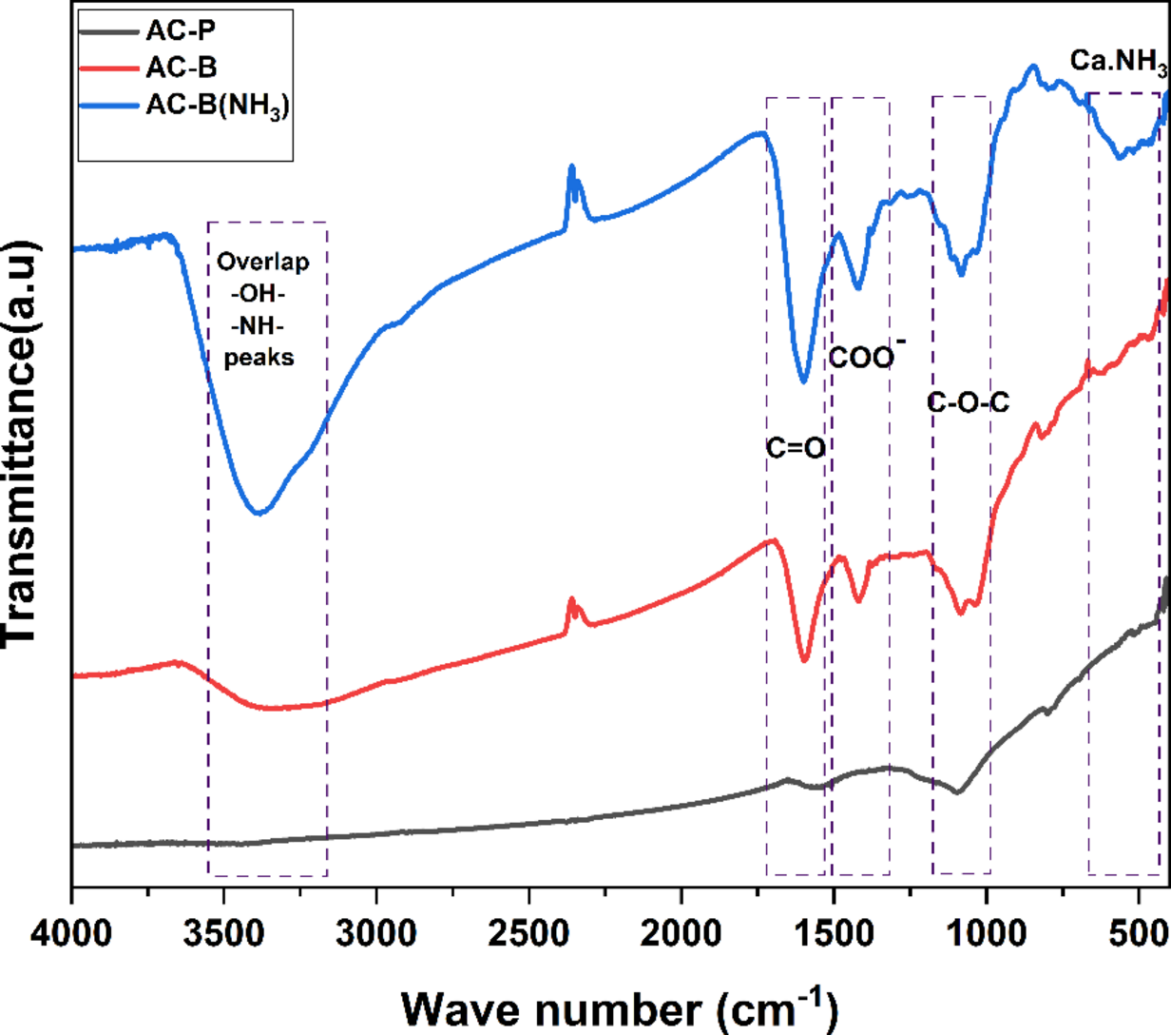
FTIR analysis of AC-B (20%) sample before, after adsorption cycle

**Fig. 11:**
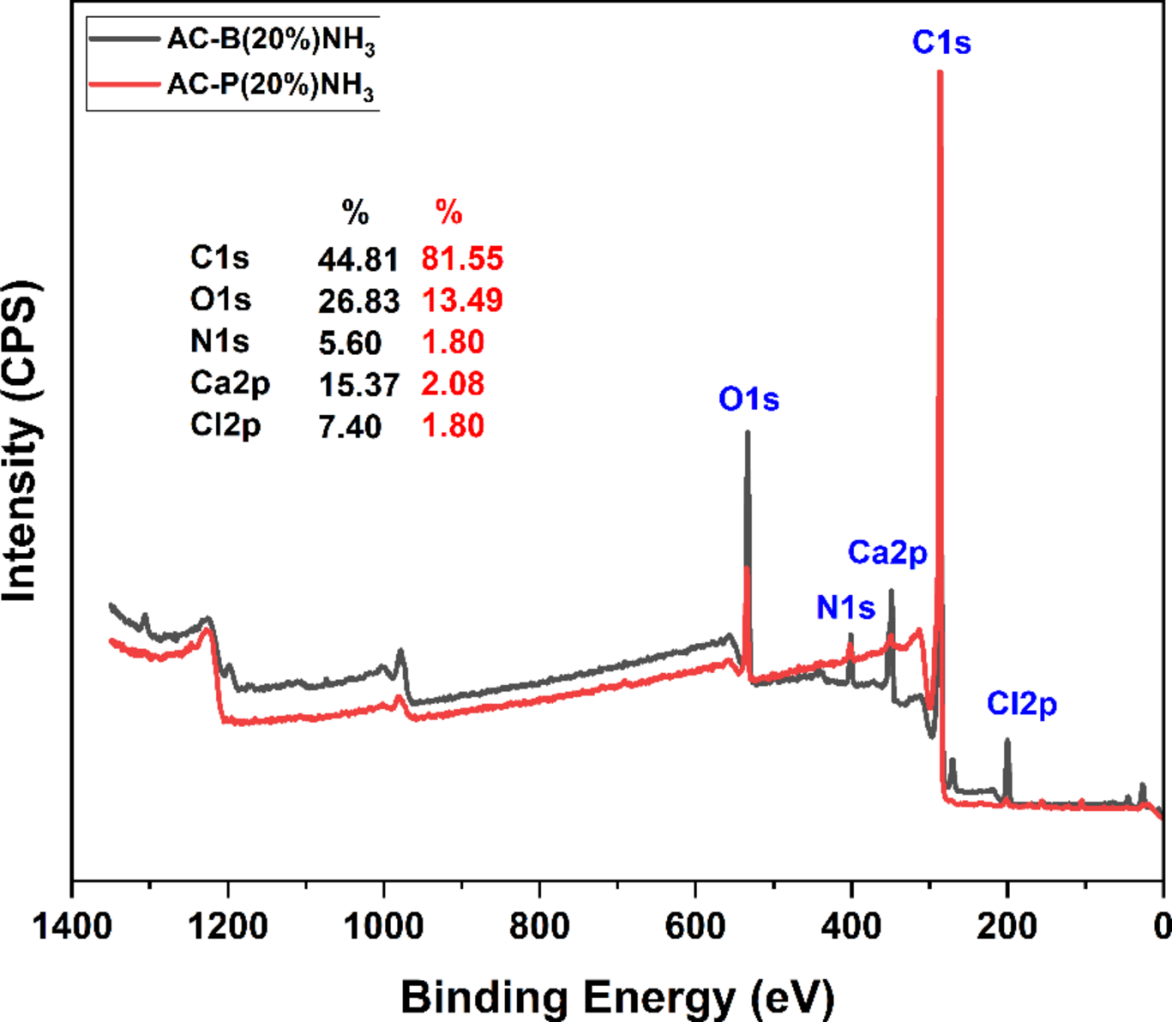
XPS survey of AC-P (20%) and AC-B (20%) samples after ammonia adsorption

**Fig. 12:**
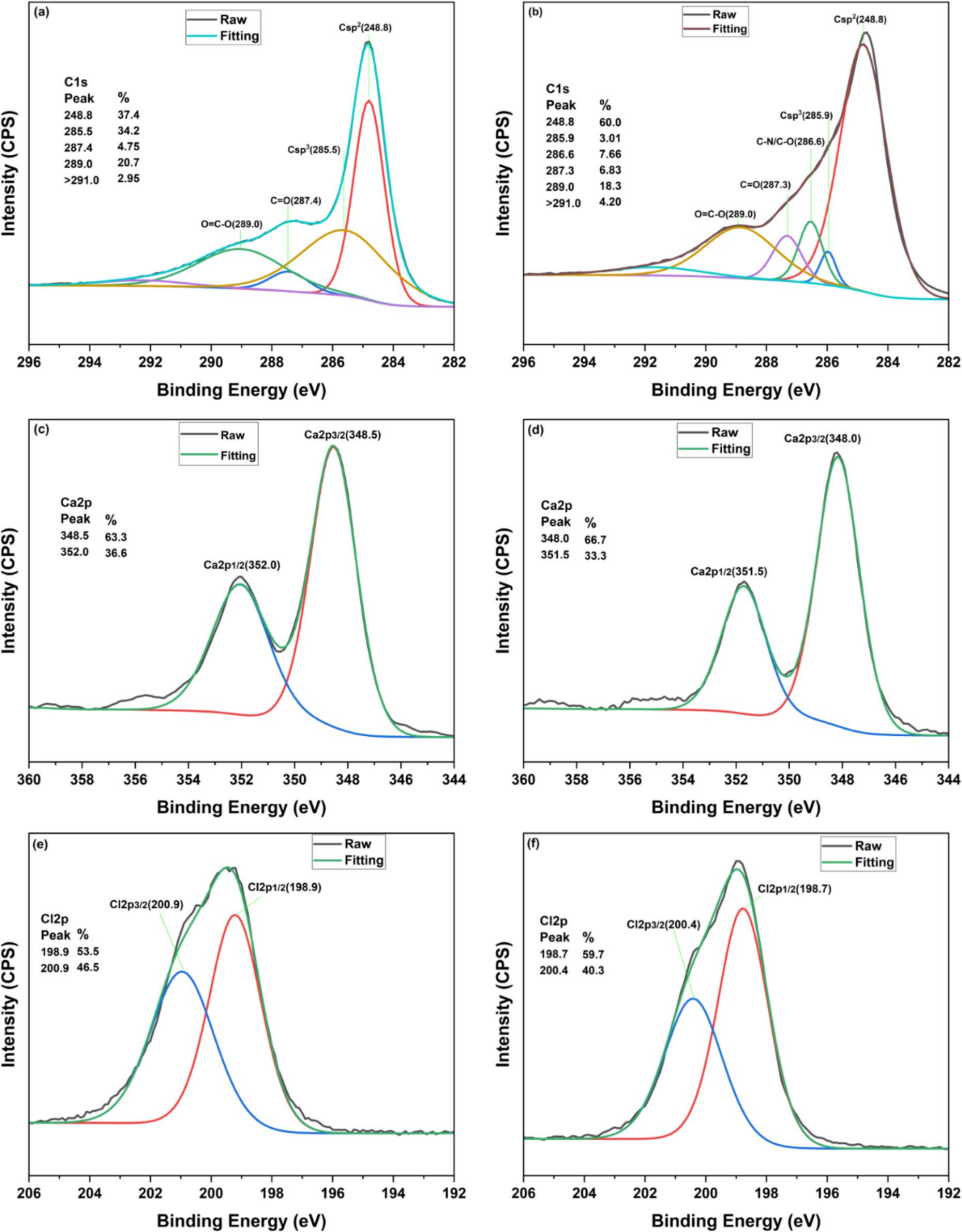
XPS analysis of AC-B (20%), before and after exposure to ammonia. XPS profile before exposure to ammonia (**a**) Carbon (**c**) Ca2p and (**e**) Cl2p. XPS profile after exposure to ammonia (**b**) carbon (**d**) Ca2p (f) Cl2p

Initially, a calcium ion interacts with a lone pair of electrons of nitrogen in ammonia to form a coordinated bond. At the same time, ammonia coordinates with calcium ions in calcium chloride to balance charge and stabilize the complex. The ammonia adsorption on bead samples (AC-B (10%), AC-B (20%), and AC-B (30%)) involved various interactions other than those discussed above. Previously, FTIR and BET analysis revealed that various functional groups such as OH, COOH, COO^-^, and C=O become abundant on the surface of beads due to alginic acid and AC. Moreover, salt got embedded inside the pores of activated carbon. This arrangement positively enhanced the interaction of ammonia gas on the surface and inside the pores of beads. To get complete insight, XRD, FTIR, and XPS analysis of the best sample (AC-B-20%) were conducted before, after one, and after the 3^rd^ ammonia adsorption cycle, represented in Figs. 9, 10,11, and 12.

Here, the XRD analysis represents the changes in the prominent peak of AC-B (20 wt%) before and after ammonia testing. The peak at 29.2° (111) has been shifted towards a lower angle (28.9°) after the 1 st and 3rd time ammonia adsorption. Furthermore, the intensity of various peaks has also been increased [96]. These modifications are clear indications that after ammonia adsorption, some changes occurred, such as shifting towards low angles, illustrating the intercalation of ammonia molecules inside AC and CaCl_2_, resulting in the expansion of the crystal lattice [97]. In previous work by A. D. Grekova et al. [98], a similar pattern was observed for BaCl_2_ + BaBr_2_ salt due to the disordering of the composite salt phase after the ammonia adsorption/desorption cycle.

To further explore this claim, FTIR analysis was conducted for the AC-B (20%) sample before and after ammonia adsorption. According to Fig. 10, a clear increment in the intensity of the peaks can be observed for the AC-B (20%) sample after ammonia adsorption. The peak near 3300 cm^−1^ indicates the overlap of -OH and -NH peaks. Here, the -OH contributions arise from alginic acid, activated carbon, and moisture in CaCl_2_, while the -NH peak confirms ammonia adsorption [47–50]. A minor peak near 2400 cm^−1^ was also observed and is attributed to background CO_2_ interference. Despite rigorous sample preparation including 24 h drying of samples and KBr in oven and rapid sample transfer this CO_2_ contribution is unavoidable and consistent with previous literature reports [47, 51–53]. Whereas the variation in the peaks around 1600 cm^−1^ and 1073 cm^−1^ highlights the presence of Lewis acid-base interaction between ammonia (Lewis base) and electron-deficient regions on AC (Lewis acid sites) [98, 99]. These sites are created by functional groups such as carbonyl and phenol, which attract electron pairs from ammonia, resulting in adsorption. Another broad peak around ~500 cm^−1^ is extremely imperative, which suggests the variation in CaCl_2_ is due to the presence of Ca.NH_3_ interactions approve the adsorption of ammonia by salt and validate the reaction mechanism discussed above. In 1980, J. Schmidt et al. [100], used the IR and FIR and investigated the formation of coordinated bond Ca(NH_3_)_n_Cl_2_ after ammonia adsorption in CaCl_2_. His work concluded that this complex formation caused perturbations to the adsorbent lattice due to the penetration of NH_3_ molecules into the bulk [100], which validates our work. Recently, X. Tian et al. [94], analyzed ammonia adsorption by calcium chloride confined in COF and confirmed the coordinating interaction of NH_3_ to Ca^2+^ together with hydrogen bond interaction between NH_3_ and Cl^−^ after ammonia adsorption.

To complement the previous discussion, the XPS analysis of selected bead (AC-B (20%)) and powdered (AC-B (20%)) samples was conducted. The XPS survey in Fig. 11 depicted a critical comparative analysis. As per peak area calculations, the AC-B (20%) sample has more presence of CaCl_2_ salt (Ca2p (15.37%) and Cl2p (7.40%)) on the surface than AC-P (20%) (Ca2p (2.08%) and Cl2p (1.08%)), in alignment with the mechanism discussed above. Subsequently, the peak for nitrogen at 400.6 eV is 1.80% for the powdered sample, which is significantly lower than the nitrogen peak at 400.56 eV, which is 5.60% for the bead sample. These peaks can be assigned to H_3_N-Ca interactions, coordinating with previous studies [94, 95]. As the XPS cannot penetrate into the sample, the surface nitrogen is due to the presence of 5.60% ammonia, affirming more ammonia adsorption by AC-B (20%) [101].

In Fig. 12 (a), (b), (c), (d), (e), and (f), the deconvolution of C1 s, Ca2p, and Cl2p spectra of the AC-B (20%) sample was conducted to evaluate the impact of surface chemistry on the ammonia adsorption mechanism. The peaks in the C1 s spectra at 287.4 and 289.0 eV show the presence of C=O and O=C-O [102–104]. The region above 291 eV can be due to carbonate compounds and π–π∗ bands [105–107]. After ammonia adsorption, the Csp^3^ peak reduced and shifted from 285.5 to 285.9 eV. This might be due to a change in the local bonding environment of Csp^3^ carbon atoms as interaction with ammonia altered the electron density around the atom and slightly increased the binding energy. Similarly, the emergence of a new peak at 286.6 is corresponding to C-N, C-O-N, C-NH, or similar interactions, supporting adsorption [102]. The differential assessment of the Ca2p XPS spectrum revealed that Ca2p^3/2^ and Ca2p^1/2^ slightly decreased from 348.5 to 352.0 eV to 348.0 to 351.5 eV. This explicitly suggested the coordinated interaction between Ca^2+^ and NH_3_, which caused the pull of the electron cloud of the Ca atom towards the N atoms [95]. Analogously, the Cl2p peaks signal decreased from 198.9, 200.9 eV to 198.7, 200.4 eV after ammonia adsorption, corroborating the hydrogen bond interactions between Cl^-^ and ammonia [92, 94].

### Impact of pH on ammonia adsorption

**Fig. 13:**
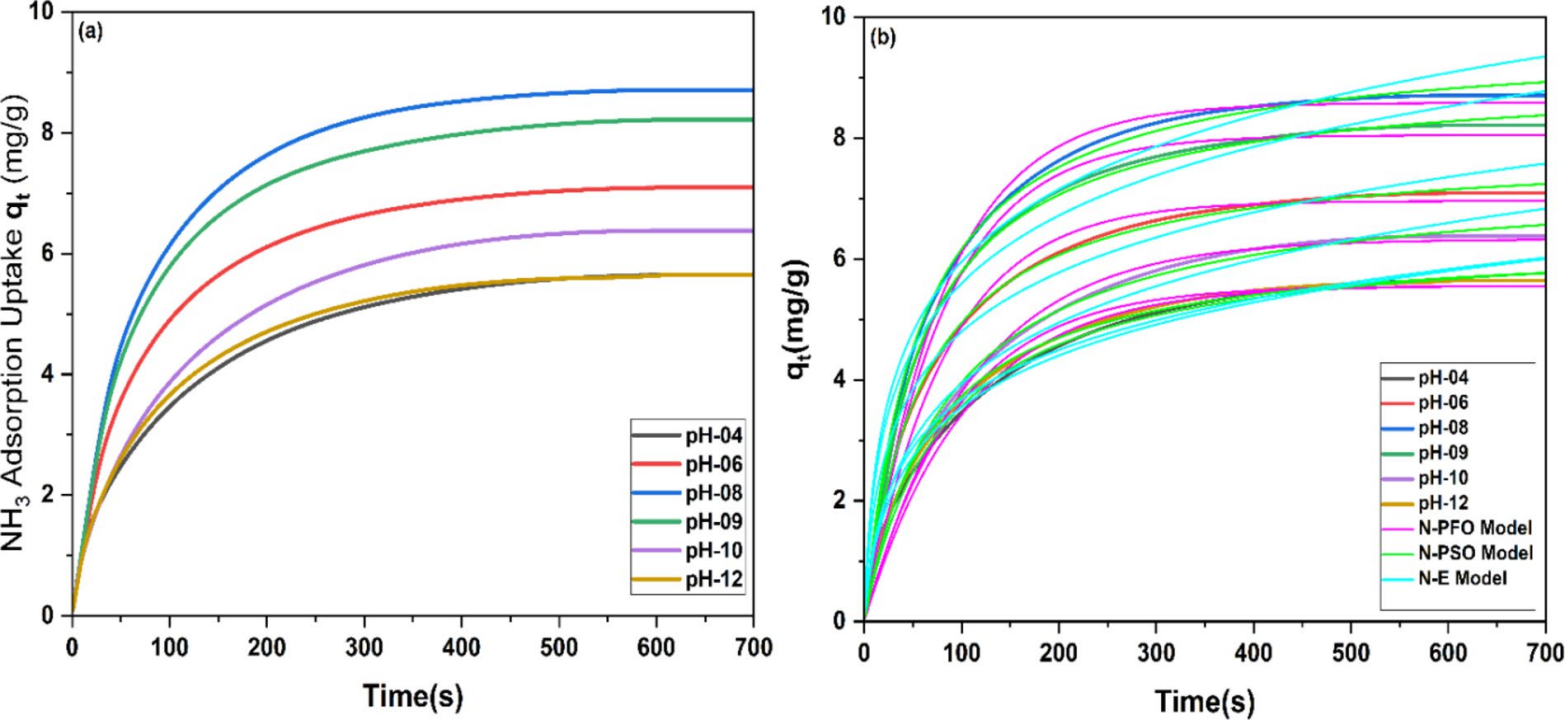
Ammonia adsorption curves and Kinetic model analysis of AC-B (20%), crosslinked at six different pH solutions (4, 6, 8, 9, 10, and 12)

To delve further into the specifics of ammonia adsorption, pH analysis was conducted by fabricating bead sample AC-B (20%) in 4, 6, 8, 9, 10, and 12 pH crosslinking solutions. The ammonia adsorption curves in Fig. 13 depict that by changing the pH, the ammonia adsorption also varies, as from 4 to 12 pH, the following values 5.62 mg/g, 7.08 mg/g, 8.70 mg/g, 6.38 mg/g, and 5.64 mg/g were measured. The trend shows that from 4 to 8 pH, ammonia adsorption increased, whereas from 9 to 12 pH, a decreasing trend was observed. To dig further, the kinetic analysis of these results was conducted by applying nonlinear PFO, PSO, and Elovich models.

According to tabulated data and curves, the N-PSO model fits very well on the experimental data with a maximum R^2^ value near 0.997 as compared to N-PFO with a maximum R^2^ value of 0.871 and an R2 value of 0.89 for the N-E model. This complements the dominance of chemical interaction over physical ones during ammonia adsorption, and apart from this, analysis of rate constants k_1_, k_2_, α, and β revealed that the adsorption rate is fast in the AC-B (20%) bead, fabricated at pH-8 as compared to other samples Table 3.

**Table 3:** Constants of Non-linear Pseudo 1^st^ order, Non-linear Pseudo 2^nd^ order and Non-Linear Elovich model

Sample (AC-B (20%))	N-PFO Model		N-PSO Model		N-E model		
	k_1_	R^2^	k_2_	R^2^	α	β	R^2^
	(s^-1^)		(g/mg.s)		(mg/g.s)	(g/mg)	
pH-04	0.0095	0.883	0.019	0.995	0.18	0.76	0.89
pH-06	0.0121	0.871	0.021	0.997	0.37	0.68	0.88
pH-08	0.0170	0.882	0.011	0.995	0.73	0.50	0.85
pH-09	0.0123	0.861	0.019	0.997	0.46	0.60	0.87
pH-10	0.0092	0.843	0.020	0.996	0.18	0.64	0.89
pH-12	0.0105	0.871	0.022	0.996	0.22	0.80	0.86

**Fig. 14:**
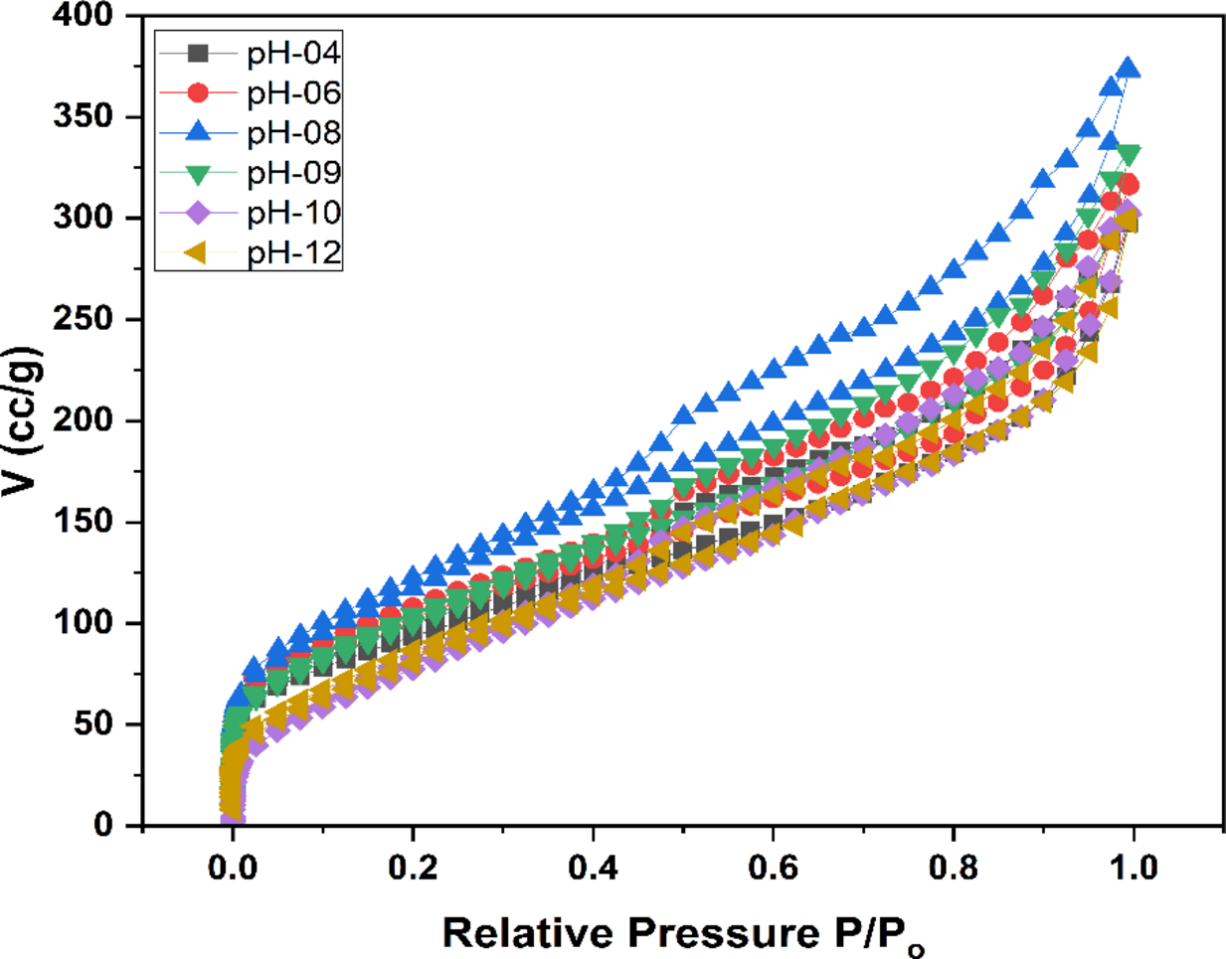
N_2_ adsorption/desorption isotherms of AC-B (20%) at different pH crosslinking, calculated from the N_2_ isotherms at −196 °C by the BET model

The BET analysis of these samples showed that the adsorption curves follow type I and type IV isotherms in IUPAC classification, consistent with the previous results. The data calculated from the BET method (Table 4) and DFT method (Table 2S and Fig. 4S) shows that as pH of dipping solution increased from 4 to 8, an augmenting trend was observed for pore volume. Conversely, as pH increased from 9 to 12, a decreasing trend in total volume was observed. To dig it further, 4 samples with pH-6, pH-8, pH-9 and pH-10 were analysed for meso and microporosity using DFT method. The figure and table show that at pH-6 to pH-8, increasing trend was observed in total pore volume, mesoporosity and microporosity. However, as we increase the pH to 9 and 10, a decreasing trend in total volume was observed, whereas a drastic drop in microporosity can be seen. The results can be discussed as per previous studies, the protonation and deprotonation of carboxyl groups on mannuronate and guluronate blocks (alginic acid) depend on their respective pKa values of 3.38 and 3.65 [108, 109]. The deprotonation of carboxylic acid began to increase as the pH of the crosslinking solution rose above its pKa value [110]. However, below that carboxylic acid is primarily protonated [109]. Here, calcium ions were highly soluble at low pH < 6, and alginic acid functional groups (COOH) were more likely to be protonated. The crosslinking was insufficient to fabricate beads with better porosity. Whereas, at pH 6–8, the carboxyl groups were partially deprotonated to COO⁻. In this range, Ca^2+^ ions remained soluble and unable to be precipitated as Ca(OH)_2_. This resulted in better crosslinking and homogeneity to form beads with improved microporosity as compared to mesoporosity. On the contrary, at high pH value of 9 - 12, the calcium ions interacted with -OH⁻ ions to form Ca(OH)_n_. This reduced the availability of Ca^2+^ to crosslink the guluronic monomers to form proper beads, as mentioned in previous work [111, 112]. There is a possibility that the precipitation of Ca(OH)_2_ has blocked or collapsed the microporosity, resulting in drastic reduction in microporosity Fig. 14.

**Table 4:** Brunauer-Emmett-Teller (BET) specific surface area (S_Bet_), pore volume (slit-like structure), and characteristic adsorption

Samples	S_Bet_	Total Pore volume (V_t_)	Avg. pore diameter (L_0_) (slit)	Characteristic adsorption energy (E_o_)
	(m^2^/g)	(cm^3^/g)	(nm)	kJ/mol
pH-12	326	0.459	2.81	15.25
pH-10	329	0.466	2.83	15.22
pH-09	379	0.512	2.70	15.40
pH-08	421	0.576	2.71	15.39
pH-06	364	0.488	2.68	15.43
PH-04	338	0.459	2.71	15.39

Now, combining adsorption analysis, kinetic model analysis, and BET method analysis, it can be concluded that here chemical interactions were prominent as compared to physical interactions, and the ammonia gas diffused from the surface of the bead towards the pores of activated carbon. The calcium chloride was present on the surface, working as a connector of guluronic acid monomers (to form the egg-box structure), and inside the AC pores, it interacted with ammonia and formed the CaCl_2_·NH_3_ complex. However, physical van der Waals interactions and hydrogen bonding also played their part in ammonia adsorption due to the presence of OH, COOH, COO^-^, and C=O bonds. The balance of all these bonds was managed till pH-8, and afterwards ammonia adsorption decreased. Owing to the fact that at high pH, excessive deprotonation caused a high number of carboxylate ions (COO^-^) in the bead, which created repulsive forces between the nitrogen group of ammonia and carboxylate ions. Most importantly, Ca^2+^ interacted with OH to form Ca(OH)_n_, which lost its activity in crosslinking and ammonia adsorption [111, 112].

### Desorption analysis of ammonia

**Fig. 15:**
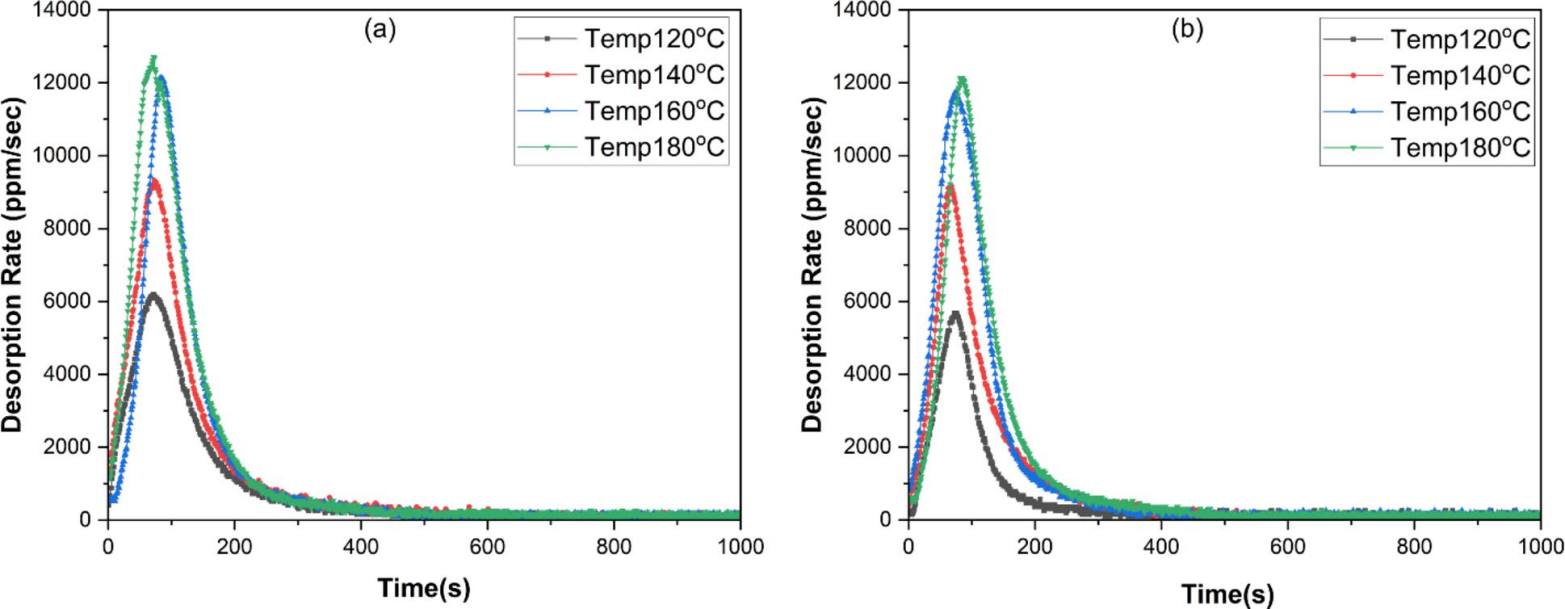
Desorption profile of (**a**) AC-B (20%), (**b**) AC-B (20%) at 120 °C, 140 °C, 160 °C, and 180 °C

The desorption analysis of AC-P (20%) and AC-B (20%) samples was conducted at four different temperatures, such as 120 °C, 140 °C, 160 °C, and 180 °C, at constant N_2_ purging flow to analyze the impact of thermal treatment on recovery and sample regeneration. The shape of all curves depicted a similar trend. Regeneration initiated as the temperature began to rise, and within 100 s, desorption reached its maximum point. It took 600 s to reach equilibrium. As illustrated in Fig. 15, the desorption rate augmented with increasing temperature. At 120 °C and 140 °C, desorption rate for AC-B (20%) was 5676 ppm/sec and 9216 ppm/sec, whereas for AC-P (20%) it remained at 6192 ppm/sec and 9320 ppm/sec. As the temperature raised to 160 °C and 180 °C, the desorption rate nearly doubled in amount to 11,663 ppm/sec and 12,133 ppm/sec for the AC-B (20%) sample and 12,105 ppm/sec and 12,679 ppm/sec for the AC-P (20%) sample. These results indicate that the desorption rate for both samples is nearly similar at 160 °C and 180 °C temperatures, suggesting comparable performance in ammonia release. However, the desorption rate in the bead sample is a little less than the powdered sample due to the presence of strong physicochemical interactions.

**Fig. 16:**
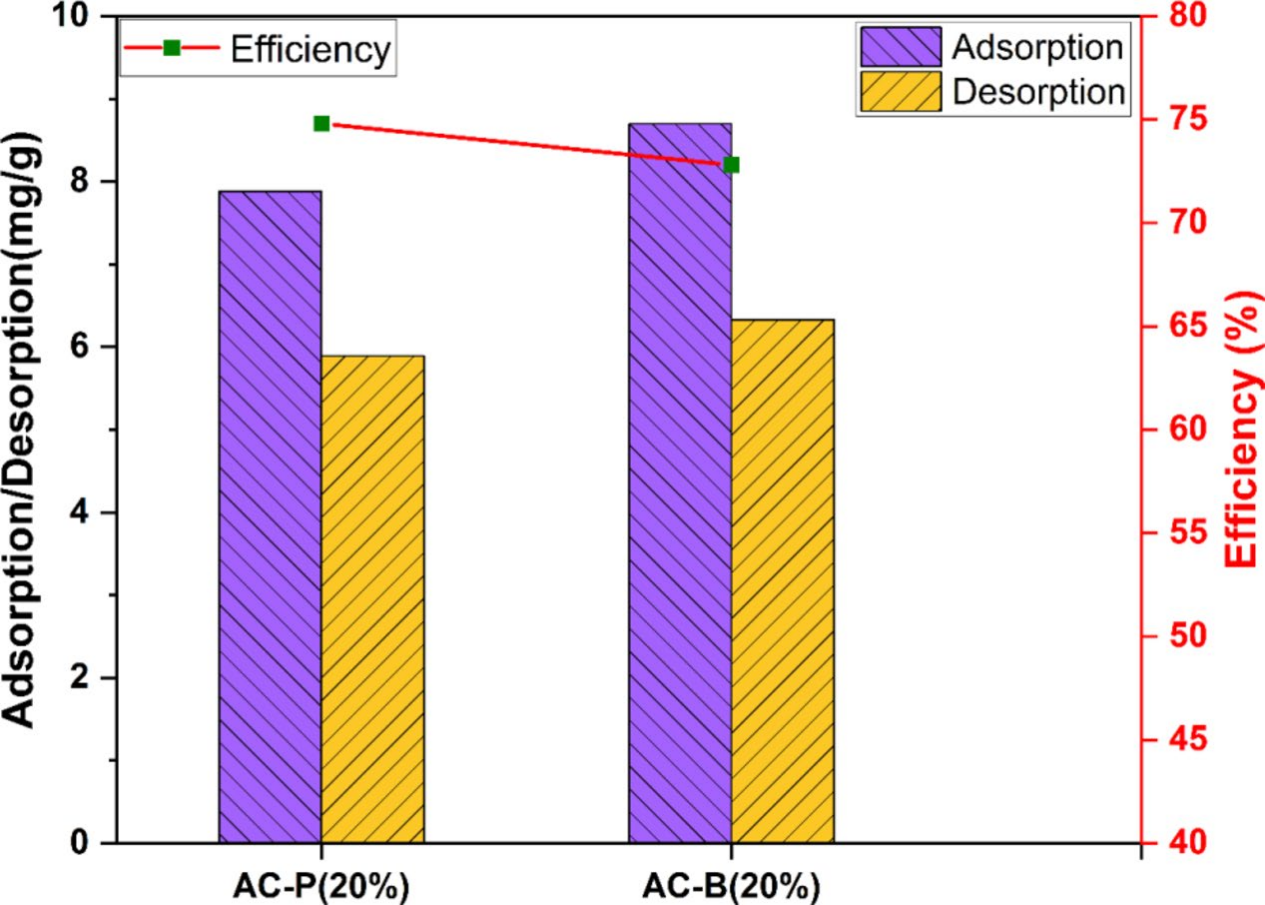
The comparison of NH_3_ absorption/desorption and regeneration efficiency of AC-B (20%) and AC-P (20%) absorbents at 180 °C

Figure 16 summarizes the adsorption, desorption, and regeneration efficiency of AC-P (20%) and AC-B (20%) samples, keeping the regeneration temperature at 180 °C. As previously mentioned, the adsorption capacity of the bead sample is more than that of the powdered sample, and the temperature range of 160 °C and 180 °C is more appropriate for desorbing ammonia. However, the desorbed concentration increased to 74.8% for AC-P (20%) and 72.8% for AC-B (20%) at 180 °C. The slightly lower desorption efficiency in the bead sample as compared to the powdered sample is due to the presence of increased binding affinity, resulting from the additional active sites provided by alginic acid. Unlike AC-P (20%), which consists solely of activated carbon and CaCl_2_, AC-B (20%) contains alginic acid that provides more defined interactions such as hydrogen bonding, Van der Waals forces, and electrostatic interactions with the adsorbate [113, 114]. This explanation is in accordance with the previously discussed adsorption mechanism.

### Stability and recyclability analysis

**Fig. 17:**
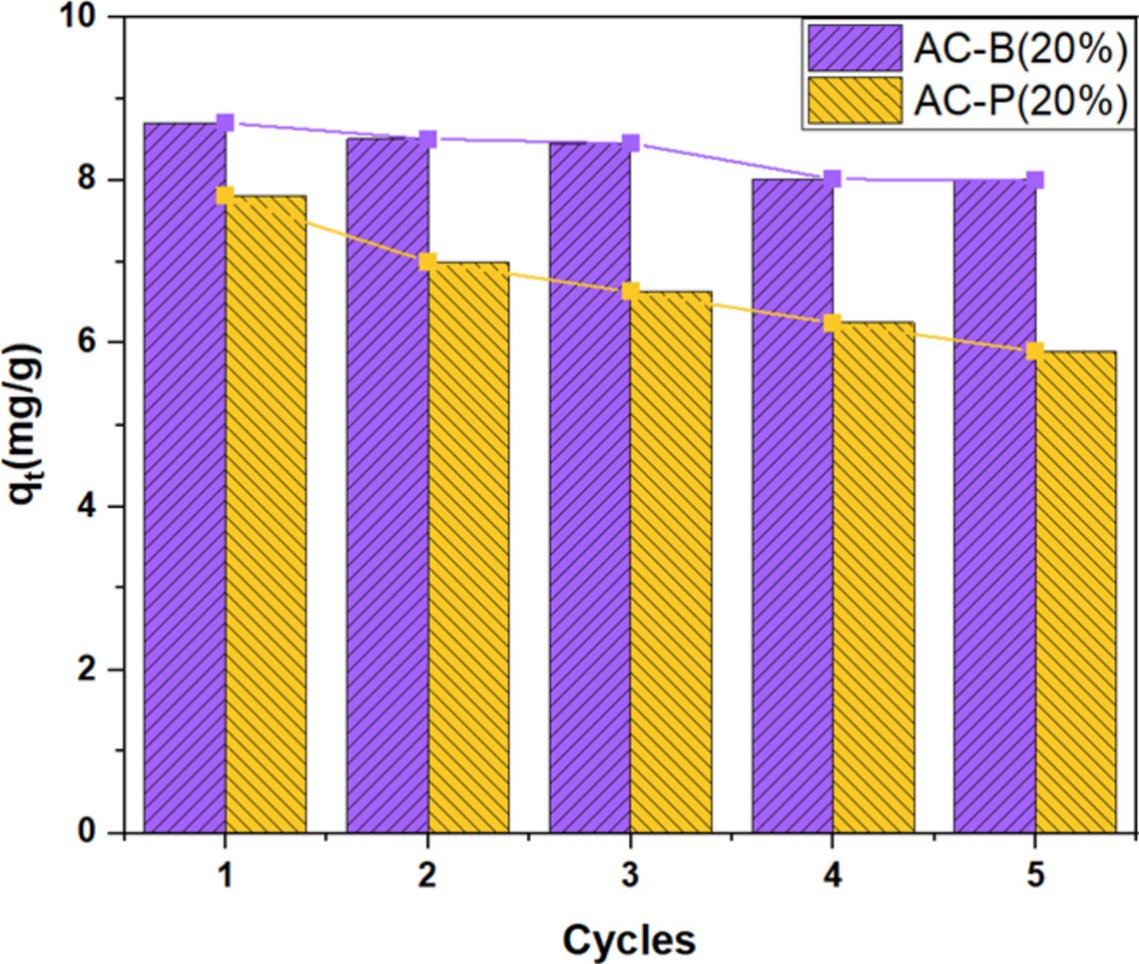
The regeneration performance test of AC-P (20%) and AC-B (20%) over five cycles at 180 °C

To evaluate the long-term stability and recyclability of the material, both samples AC-B (20%) and AC-P (20%) were tested five times. The ammonia adsorption was conducted following the same steps as previously discussed in this study. The regeneration of the samples was conducted at 180 °C. From Fig. 17, it can be observed that in the first three times, the AC-B (20%) showed nearly the same ammonia adsorption; however, a drop was observed on fourth and fifth cycles. Whereas, in the case of AC-P (20%) at every test adsorption quantity dropped sharply. To analyze these results some imperative points need to be considered as the AC-B (20%) is a bead and this structuring caused the beads to be a more rigid and stable structure, which maintained the porosity and adsorption sites. This might also have caused the binding of CaCl_2_ salt, resulting in the preservation of adsorption sites. However, after the third cycle, adsorption sites might become blocked, whereas, the structure of beads was maintained, and the total drop in adsorption after five cycles is 8.7%. In the case of the AC-P (20%) sample, after every cycle a drastic drop in adsorption was observed, and it might be because of agglomeration, which caused a reduction in mass transfer and led to a drop in ammonia adsorption by 24%. This performance degradation is attributed to structural agglomeration variations in gas flow and temperature fluctuations, which hindered mass transfer and reduced access to active sites, while the bead structure remained intact. Despite the minimal difference in efficiency, the overall performance and stability of AC-B (20%) signifies that bead samples are a better option due to their structural and increased binding affinity over powdered samples for ammonia storage.

### Economic feasibility analysis of fabricated samples

In this study, activated carbon, calcium chloride, and alginic acid were used to fabricate samples for ammonia adsorption. The economic feasibility of these materials is high compared to previously studied alternatives for the same application. For example, activated carbon can be purchased for around $5– $10 per kilogram (kg), making it suitable for large-scale applications. Similarly, CaCl_2_, a common salt used in various industries, costs around $10– $15 per kg. Lastly, alginic acid, a green material, generally costs around $5– $15 per kg. In contrast, previous studies have shown that various MOFs, such as MFM-300, UIO-66, MIL-160, MOF-5, and MOF-177 have been used to adsorb ammonia [115, 116]; however, their estimated cost is around $500– $10,000 per kg, which reduces their economic viability and recyclability at an industrial scale [117]. In several instances, the unavailability of these specific MOFs has persuaded researchers to fabricate them in the laboratory, but the high raw material cost and the complicated, energy-intensive synthesis procedures still make them economically unfeasible. Moreover, the materials used in this study are environmentally friendly, whereas various MOFs have a high environmental footprint and are fabricated using toxic solvents [118]. Thus, the composite materials used in this study offer advantages over expensive materials due to their low cost, robust structural performance, and environmentally friendly characteristics.

## Conclusion

In conclusion, the synergetic impacts of structured and powdered calcium chloride-based activated carbon composites were comparatively analyzed for ammonia adsorption. In this work, composites were fabricated and examined quantitatively/qualitatively using characterization techniques such as SEM, XRD, FTIR, XPS, BET, and DFT. The ammonia adsorption studies were conducted on the designed rig, and the impact of porosity, salt distribution, and pH-controlled crosslinking (powdered and bead samples) on the ammonia adsorption mechanism was explored. To complement these findings, further FTIR, XRD, and XPS analysis was conducted before and after ammonia adsorption. As per the results, the bead sample crosslinked at pH-8 with 20% CaCl_2_ (AC-B(20%)) has adsorbed ammonia 8.77 mg/g, double the amount of 4.04 mg/g as compared to the pure AC powder sample and higher than the 20% CaCl_2_-impregnated AC sample (AC-P(20%)), due to better porosity, optimized salt distribution on the surface, inside pores, and physicochemical interactions such as Van der Waals, hydrogen bonding, and CaCl_2_.NH_3_ complex formation. The kinetic analysis of experimental results was conducted using the non-linear PFO, PSO, and Elovich models, with the N-PSO model fitting very well, validating the adsorption mechanism and also depicting the rate of mass transfer as high in the AC-B (20%) bead sample as compared to the powder sample. The regeneration analysis revealed that bead and powdered samples can be regenerated, 70 to 73% in the temperature range of 160–180 °C. However, the recyclability analysis revealed that the bead sample retained its structure, maintaining adsorption with slight drop in performance, whereas the adsorption capacity of the powdered sample decreased by 24% due to agglomeration. Lastly, the economic feasibility analysis proved that the composite materials are attractive due to their cost-effectiveness, better structural integrity, and eco-friendly characteristics. These results demonstrate that structuring activated carbon containing CaCl_2_ in the form of beads can enhance ammonia adsorption while offering improved handling and storage. Future studies can be focused further to develop new types of structured composites, using similar approaches and investigating their potential utility in energy storage applications.

## Electronic supplementary material

Below is the link to the electronic supplementary material.

Supplementary Material 1

## Data Availability

The datasets used and analyzed during the current research study will be made available on reasonable request.
