# Supplementary material for: Synergistic effects of structured and powdered Calcium Chloride-Activated Carbon composites on Ammonia adsorption: the role of salt distribution and pH-controlled crosslinking
Source: Emergent Mater. 2025 May 8;8(6):5217–40. doi: 10.1007/s42247-025-01106-8 (PMC12528320; doi:10.1007/s42247-025-01106-8)
Supplement: Supplementary file 1 — Supplementary Material 1 [file 42247_2025_1106_MOESM1_ESM.docx]

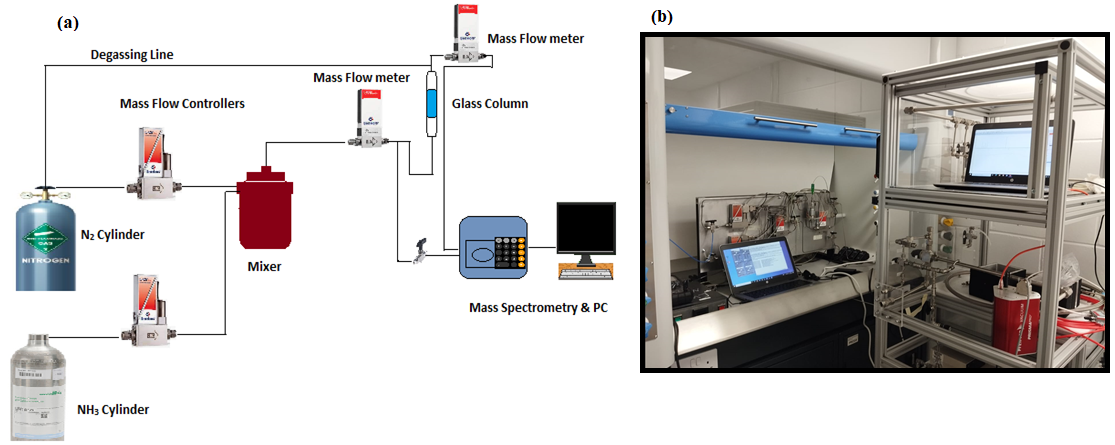


**Fig. 1S (a) Schematic representation of ammonia adsorption rig, (b) Designed equipment in the lab**

**
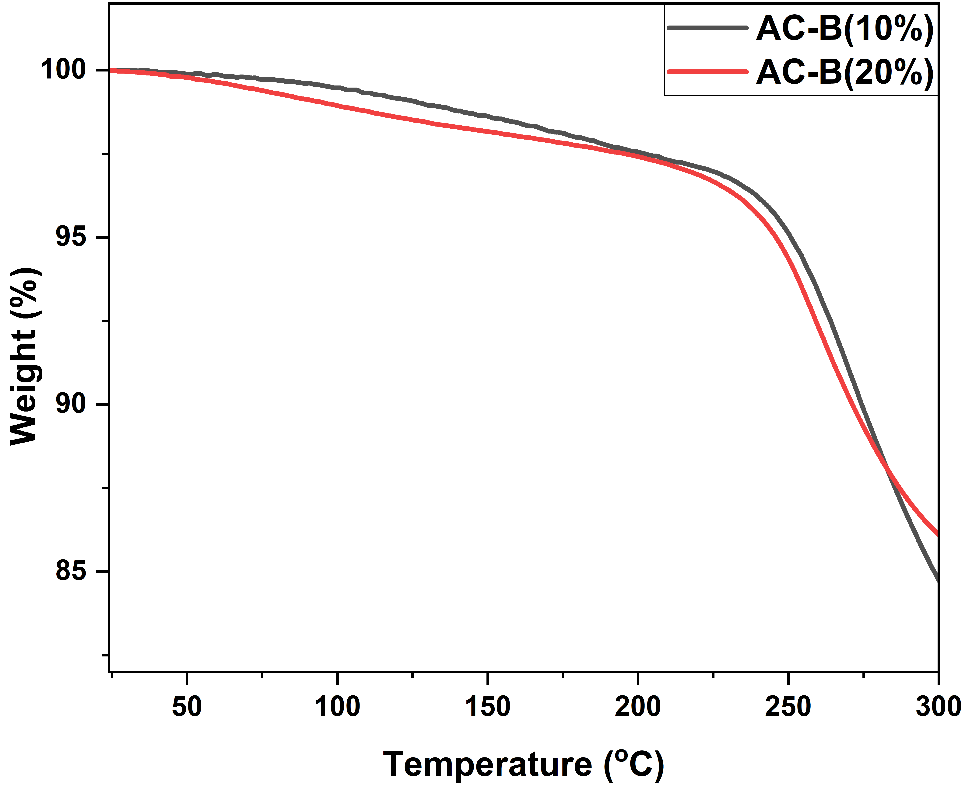
**

**Fig. 2S Thermogravimetric Analysis (TGA) of AC-B (20%) and AC-B (10%)**

**
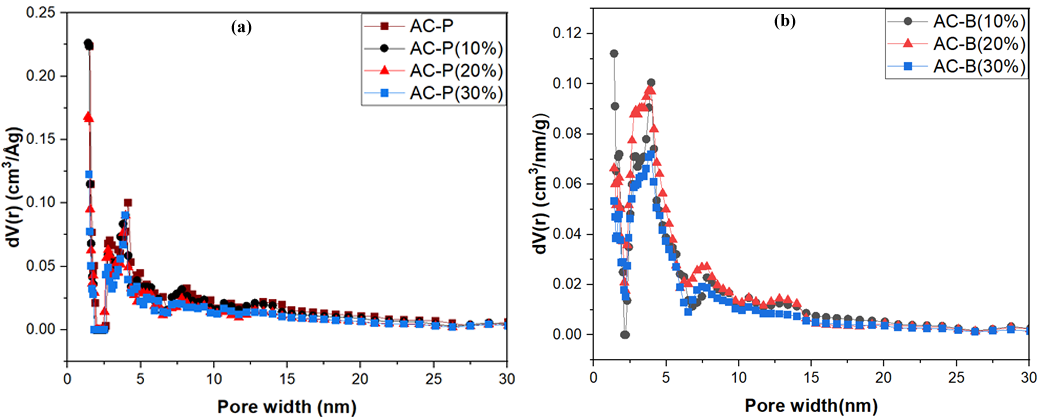
**

**Fig. 3S (a) Pore size distribution (PSD) of powder and (b) bead samples, calculated from the N_2_ isotherms at −196 °C by the DFT method**

**
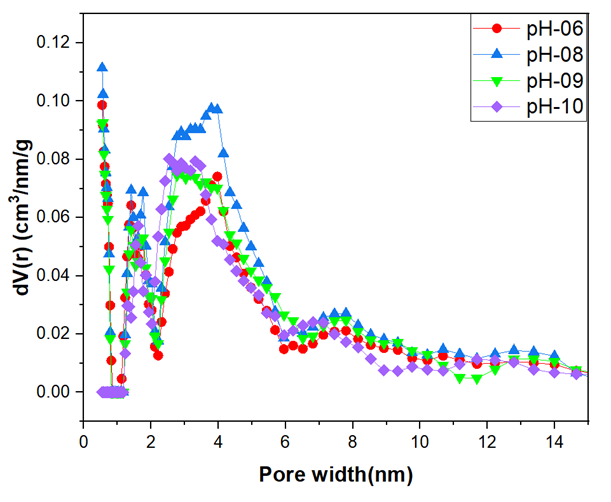
**

**Fig. 4S Pore size distribution (PSD) of AC-B (20%) at different pH crosslinking, calculated from the N_2_ isotherms at −196 °C by the DFT method**

**Table 1S. Pore volume, micropore and mesopore pore volume of Powdered and bead samples using DFT model**

| Sample | Pore volume (cm^3^/g) | Micropore volume (cm^3^/g) | Mesopore volume  (cm^3^/g) |
| --- | --- | --- | --- |
| AC-P | 0.689 | 0.194 | 0.495 |
| AC-P (10%) | 0.658 | 0.189 | 0.469 |
| AC-P (20%) | 0.517 | 0.146 | 0.371 |
| AC-P (30%) | 0.457 | 0.106 | 0.351 |
| AC-B (10%) | 0.509 | 0.104 | 0.405 |
| AC-B (20%) | 0.520 | 0.086 | 0.434 |
| AC-B (30%) | 0.310 | 0.048 | 0.262 |

**Table 2S. Pore volume, micropore and mesopore pore volume of bead samples (at various pH) using DFT model**

| Samples  AC-B (20%) | Pore volume (cm^3^/g) | Micropore volume (cm^3^/g) | Mesopore volume (cm^3^/g) |
| --- | --- | --- | --- |
| pH-10 | 0.421 | 0.0235 | 0.397 |
| pH-09 | 0.457 | 0.0648 | 0.392 |
| pH-08 | 0.520 | 0.0863 | 0.434 |
| pH-06 | 0.438 | 0.0844 | 0.353 |
